# Phylogenomics of the olive tree (*Olea europaea*) reveals the relative contribution of ancient allo- and autopolyploidization events

**DOI:** 10.1186/s12915-018-0482-y

**Published:** 2018-01-25

**Authors:** Irene Julca, Marina Marcet-Houben, Pablo Vargas, Toni Gabaldón

**Affiliations:** 1 Centre for Genomic Regulation (CRG), The Barcelona Institute of Science and Technology, Dr. Aiguader 88, Barcelona, 08003 Spain; 2 Universitat Pompeu Fabra (UPF), 08003 Barcelona, Spain; 3 Universitat Autònoma de Barcelona (UAB), 08193 Barcelona, Spain; 4 Real Jardín Botánico de Madrid (CSIC-RJB), 28014 Madrid, Spain; 5 ICREA, Pg. Lluís Companys 23, 08010 Barcelona, Spain

**Keywords:** Olive, Lamiales, Polyploidy, Phylogenomics, Hybridization

## Abstract

**Background:**

Polyploidization is one of the major evolutionary processes that shape eukaryotic genomes, being particularly common in plants. Polyploids can arise through direct genome doubling within a species (autopolyploidization) or through the merging of genomes from distinct species after hybridization (allopolyploidization). The relative contribution of both mechanisms in plant evolution is debated. Here we used phylogenomics to dissect the tempo and mode of duplications in the genome of the olive tree (*Olea europaea*), one of the first domesticated Mediterranean fruit trees.

**Results:**

Our results depict a complex scenario involving at least three past polyploidization events, of which two—at the bases of the family Oleaceae and the tribe Oleeae, respectively—are likely to be the result of ancient allopolyploidization. A more recent polyploidization involves specifically the olive tree and relatives.

**Conclusion:**

Our results show the power of phylogenomics to distinguish between allo- and auto polyploidization events and clarify the contributions of duplications in the evolutionary history of the olive tree.

**Electronic supplementary material:**

The online version of this article (10.1186/s12915-018-0482-y) contains supplementary material, which is available to authorized users.

## Background

The duplication of the entire genetic complement—a process known as polyploidization or whole-genome duplication (WGD)—is one of the most drastic events that can shape eukaryotic genomes [1]. Polyploidization can be a trigger for speciation [2], and can result in major phenotypic changes driving adaptation [3]. This phenomenon is particularly relevant in plants, where it is considered a key speciation mechanism [4, 5], and where the list of described polyploidizations grows in parallel with the sequencing of new genomes [6–11]. Polyploidization in plants has been a common source of genetic diversity and evolutionary novelty, and is in part responsible for variations in gene content among species [3, 4, 12]. Importantly, this process seems to have provided plants with traits that make them prone to domestication [13], and many major crop species, including wheat, maize, and potato, are polyploids [6, 10, 14].

Polyploidization can take place through two main mechanisms: autopolyploidization and allopolyploidization. Autopolyploidization is the doubling of a genome within a species, and thus, resulting polyploids initially carry nearly identical copies of the same genome [2]. Allopolyploids, also known as polyploid hybrids, originate from the fusion of the genomic complements from two different species followed by genome doubling. This genome duplication can enable proper pairing between homologous chromosomes and restore offspring fertility [15–17]. This mechanism has been described as the fastest (one generation) and most pervasive speciation process in plants [18, 19]. Hence, allopolyploids harbor chimeric genomes from the start, with divergences reflecting those existing between the crossed species.

Elucidating the exact number and type of past polyploidization events from extant genomes is challenging. This is partly because, after polyploidization, the genome progressively returns to a diploid state [4, 20]. This so-called diploidization is attained through chromosome fusion or loss, (retro)transposon mobility, repetitive DNA loss, and gene loss, sometimes resulting in a relatively fast reduction of genome size [21]. For instance, *Sorghum bicolor* (sorghum) and *Zea mays* (maize) have the same number of chromosomes, even though maize underwent WGD since their divergence (~11.9 MyA) [22]. Similar examples of a rapid reduction of the number of chromosomes after polyploidization can be found in the family Brassicaceae [21]. Hence, chromosome number can be used to estimate the existence of polyploidization events, but it is not a precise indicator of the number or type of such events. Of note, it has been proposed that the nature of rearrangements and the number of losses may differ following auto- and allopolyploidization events, because in autopolyploids, in contrast to allopolyploids, the recurrent random assortment of chromosomes may select against deletions of duplicated genes, which would lead to gametes lacking a complete gene set [23].

Gene order (also known as synteny) is often used to assess past polyploidizations, generally by comparing the purported polyploid genome to a related non-duplicated genome. However, this approach requires well-assembled genomes, and its power is limited for ancient events, as the signal is blurred by the accumulation of genome rearrangements over time. Finally, phylogenomics provides an alternative approach to studying the history of polyploidizations. In particular, a topological analysis of phylomes, which are complete collections of gene evolutionary histories, has helped to uncover ancient polyploidization (paleoploidization) events [12, 24–27]. Recently, phylome analysis was instrumental in distinguishing between ancient auto- and allopolyploidization in yeast [28]. Such analyses compare topological patterns observed in gene trees and their frequencies, with the expected topologies resulting from auto- and allopolyploidization scenarios followed by gene loss. Hybridization involves non-vertical patterns of inheritance that can result in the preponderance of anomalous gene tree topologies. For instance, in the above mentioned yeast study [28], the topologies of paralogous gene families revealed that often each paralogous set of genes had orthologs only in species from one of two different yeast clades, suggesting allopolyploidization between these two clades.

The olive tree (*Olea europaea* subsp. *europaea* var. *europaea*) is one of the most important fruit trees cultivated in the Mediterranean basin [29]. It belongs to the family Oleaceae (order Lamiales). Despite the large number of families in the order Lamiales (24) [30], with the olive tree (*Olea europaea*) as the taxonomic type species, only eight families have at least one species with public genome sequences. The family Oleaceae is one of the first lineages that diverged within the Lamiales [31] and is composed of five tribes: Fontanesieae, Forsythieae, Myxopyreae, Jasmineae, and Oleeae. The last tribe is a large group that is further divided into four subtribes (Ligustrinae, Schreberinae, Fraxininae, and Oleinae) [32, 33]. The genus *Olea* belongs to the subtribe Oleinae and includes approximately 40 taxa [34]. *O. europaea* is divided into six subspecies: *europaea*, *laperrinei*, *guanchica*, *maroccana*, *cerasiformis*, and *cuspidata* [32, 35]. The subsp. *europaea* is further subdivided into two taxonomic varieties: var. *sylvestris*, also named oleaster, which encompasses the wild forms of the olive tree, and var. *europaea*, which comprises cultivated forms [32]. Despite the large number of species in the subtribe Oleinae, only two olive genomes are currently available [36, 37]. The genome of *O. europaea* has a diploid size of 1.32 Gb distributed in 46 chromosomes (2*n*). To date, polyploids have been described within *O. europaea* as a recent polyploid (neoployploid) series (2×, 4×, and 6×) based on chromosome counting, flow cytometry, and molecular markers of living trees [29]. However, little is known about paleopolyploidizations in the olive tree and relatives. One of the analyses performed on the reference olive genome [36] revealed an increased gene content compared to other Lamiales. This very much suggests the existence of at least one past polyploidization event since the olive tree diverged from other sequenced Lamiales [36]. The sequencing of the genome of *Fraxinus excelsior* [38] and the second genome of *Olea europaea* (var. *sylvestris*) [37] confirmed the presence of at least one, possibly two, common WGDs [39]. Still, it is as yet unclear whether these events represent auto- or allopolyploidization events. To clarify this puzzle, we performed a phylogenomic analysis of the genomes of *O. europaea* and relatives.

## Results and discussion

### Gene order analysis confirms multiple polyploidizations in the Lamiales

A standard approach to confirming polyploidization relies on finding conserved syntenic paralogous blocks. We searched duplicated genomic regions in the olive genome using CoGe tools [39]. Our results revealed numerous duplicated syntenic regions, which supports the existence of polyploidization events (Additional file 1: Figure S1a). We then calculated the syntenic depth of the olive genome. Syntenic depth is a measure of the number of regions in the genome of interest that are syntenic to a given region in a reference genome (see “Methods”). In the absence of a WGD, the comparison between two genomes should result in most genes having a syntenic depth of 1, indicating a low number of duplicated regions. In contrast, polyploidizations will be apparent in the form of many genes having higher syntenic depths (i.e., a peak of syntenic depth of 2 for a single WGD compared to the reference genome). Diploidization events that occur after the polyploidization will erase part of the signal, so it is not surprising to find a mix of different depths (i.e., three rounds of WGD may initially result in syntenic depths peaking at 8 = 2 × 2 × 2, but subsequent gene losses will blur this peak toward lower values of syntenic depths). As a reference for our analysis, we used *Coffea canephora.* This species belongs to the order Gentianales and, given the presence of duplications among all sequenced Lamiales species, *C. canephora* is the closest non-duplicated reference genome [40]. As a control, we performed a similar analysis between *C. canephora* and *Sesamum indicum*, a Lamiales species known to have undergone a single WGD [41]. We also included *F. excelsior* (Oleaceae) in the comparison as the closest fully sequenced relative of olive. Our analyses (Additional file 1: Figure S1b) revealed contrasting patterns between the three species. The *Sesamum–Coffea* comparisons revealed a single peak in the frequency distribution of syntenic depths at a value of 2, consistent with the reported single WGD [41]. In contrast, there was no such clear peak in the above mentioned *Olea–Coffea* or *Fraxinus–Coffea* comparisons, but rather a similarly high number of regions of depth 1 to 6, and 1 to 4, respectively. These results indicate the presence of multiple polyploidization events in the lineages leading to *O. europaea* and *F. excelsior*. Moreover, the comparatively higher values of syntenic depth in *O. europaea* suggest this species may have undergone more polyploidization events than *F. excelsior*.

### The olive phylome

**Fig. 1:**
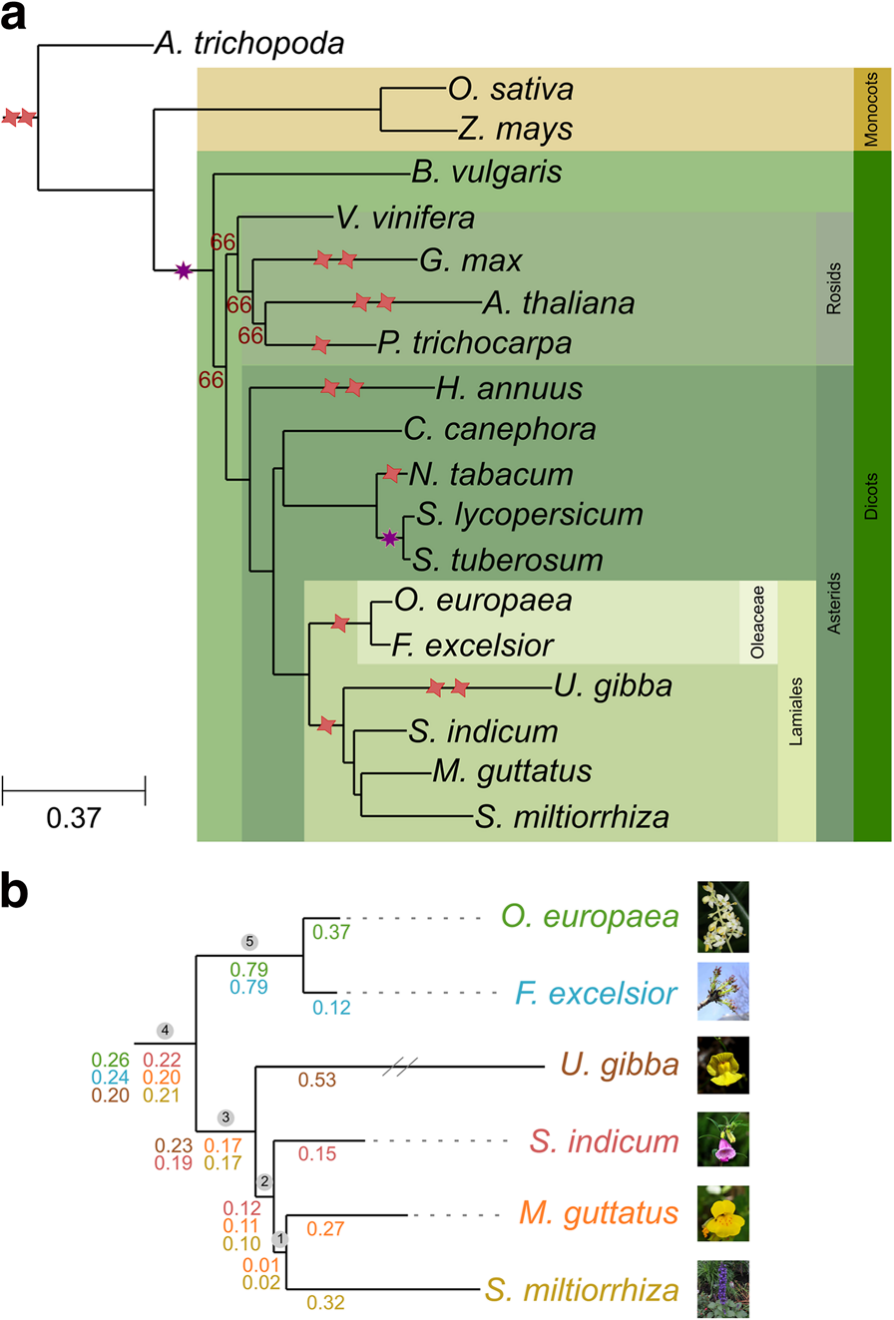
Species trees. **a** Evolutionary relationships among 19 plant species used in this study. All bootstrap values that are not shown in the graph are maximal (100). Red stars represent whole-genome duplication events and purple stars represent whole-genome triplication events. These events represent those already known at the start of this study with their proposed phylogenetic positions as described in the literature. **b** Zoom-in to the Lamiales clade. Numbers in a circle on top of internal nodes represent the node names as referred to in the text, and numbers below each branch are duplication frequencies calculated for each phylome. Each phylome and their corresponding duplication frequencies is colored differently: *O. europaea* is green, *F. excelsior* is light blue, *U. gibba* is brown, *S. indicum* is red, *M. guttatus* is orange, and *S. miltiorrhiza* is yellow

To elucidate the evolutionary history of *O. europaea* genes and compare it to that of related plants, we reconstructed the phylomes [42] of this species and those of five other Lamiales with available genome sequences (*F. excelsior*, *Mimulus guttatus*, *S. indicum*, *Utricularia gibba*, and *Salvia miltiorrhiza*). Phylomes are complete sets of gene phylogenies representing the evolutionary histories of all genes encoded in a genome of interest. The four previously published non-Oleaceae genomes are known to share a polyploidization [43] and thus, their inclusion in our analysis may help to clarify whether that polyploidization also affected the olive lineage. These phylomes are available in the PhylomeDB database [44] (see Additional file 2: Table S1 for details). We reconstructed the evolutionary relationships of the considered species using a concatenated approach with 215 widespread single-copy orthologs (Fig. 1a), which yielded congruent results with previous analyses [45, 46]. The currently proposed polyploidization events are depicted in Fig. 1a. We scanned the phylomes to infer orthologs and paralogs, and date duplication events (see “Methods”). Using relative dating of gene duplications [47], we mapped them to the corresponding clades in the species tree. Functional analyses suggest that phosphatidylinositol activity, recognition of pollen, terpene activity, gibberellin metabolism, and stress response are annotations enriched among genes duplicated in several of the nodes in the species tree (see Additional file 2: Table S2). We calculated the duplication frequency for each marked node in Fig. 1b. Four internal branches (nodes 2 to 5) and all terminal branches had high duplication frequencies (Fig. 1b). Of the terminal branches, the two highest duplication frequencies corresponded to that of *U. gibba* (0.53 duplications/gene), for which two recent WGDs have been proposed [43], and to *O. europaea* (0.37). Altogether, these analyses indicate that the lineage leading to the olive tree shows three differentiated waves of massive gene duplications: (i) one preceding the diversification of the sequenced Lamiales (node 4), (ii) another one at the base of the family Oleaceae and shared with *F. excelsior* (node 5), and (iii) a more recent one specific to the olive lineage.

### Phylogenetic analysis reveals ancient allopolyploidization in Lamiales

We focused on the duplication peaks at the internal branches 2, 3, and 4 in Lamiales (Fig. 1b). A polyploidization event has been previously described within Lamiales [48], although that study could not clarify whether the event was shared or not with Oleaceae species. Thus, the previous event could correspond to node 3 (not shared with Oleaceae) or node 4 (shared with Oleaceae). The peak at node 2, which has not previously been described, can be explained because the carnivorous plant *U. gibba*, despite the two recent WGDs, has a reduced genome resulting from massive gene loss [43]. Indeed, for duplications that occurred at node 3, loss of all the duplicated paralogs in *U. gibba* would lead to mapping to node 2. Supporting this scenario is the finding that, when excluding orphan genes, only 51% of *S. indicum* genes have orthologs in *U. gibba* (see Additional file 3: Figure S2), compared to 76% when comparing *S. indicum* to *M. guttatus* (see Additional file 3: Figure S2). To test this scenario further, we examined trees in the *S. indicum* phylome with node 2 duplications and counted how many of them included *U. gibba* homologs within the Lamiales clade. Only 20.7% of such trees fulfilled that pattern, further supporting that duplications that mapped to node 2 mostly result from duplications that had occurred at node 3 followed by gene loss in *U. gibba*.

A similar scenario could explain duplications at node 3, if massive loss had occurred in *O. europaea* and *F. excelsior*. However, these two species do not have reduced genomes (Additional file 3: Figure S2)*.* In addition, when scanning *S. indicum* phylome trees with either a duplication at node 2 or at node 3, homologs of *O. europaea* or *F. excelsior* could be found in 83.0% of them. Therefore, in this case, losses specific to Oleaceae cannot explain the duplication peak at node 3. This leads to the conclusion that at least two independent polyploidizations took place in the Lamiales*:* one corresponds to the previously described event [43] preceding the divergence of *M. guttatus* and *U. gibba* (node 3), and the other, congruent with a more ancestral event (node 4) preceding the divergence between Oleaceae and the other non-Oleaceae Lamiales species included in this study.

**Fig. 2:**
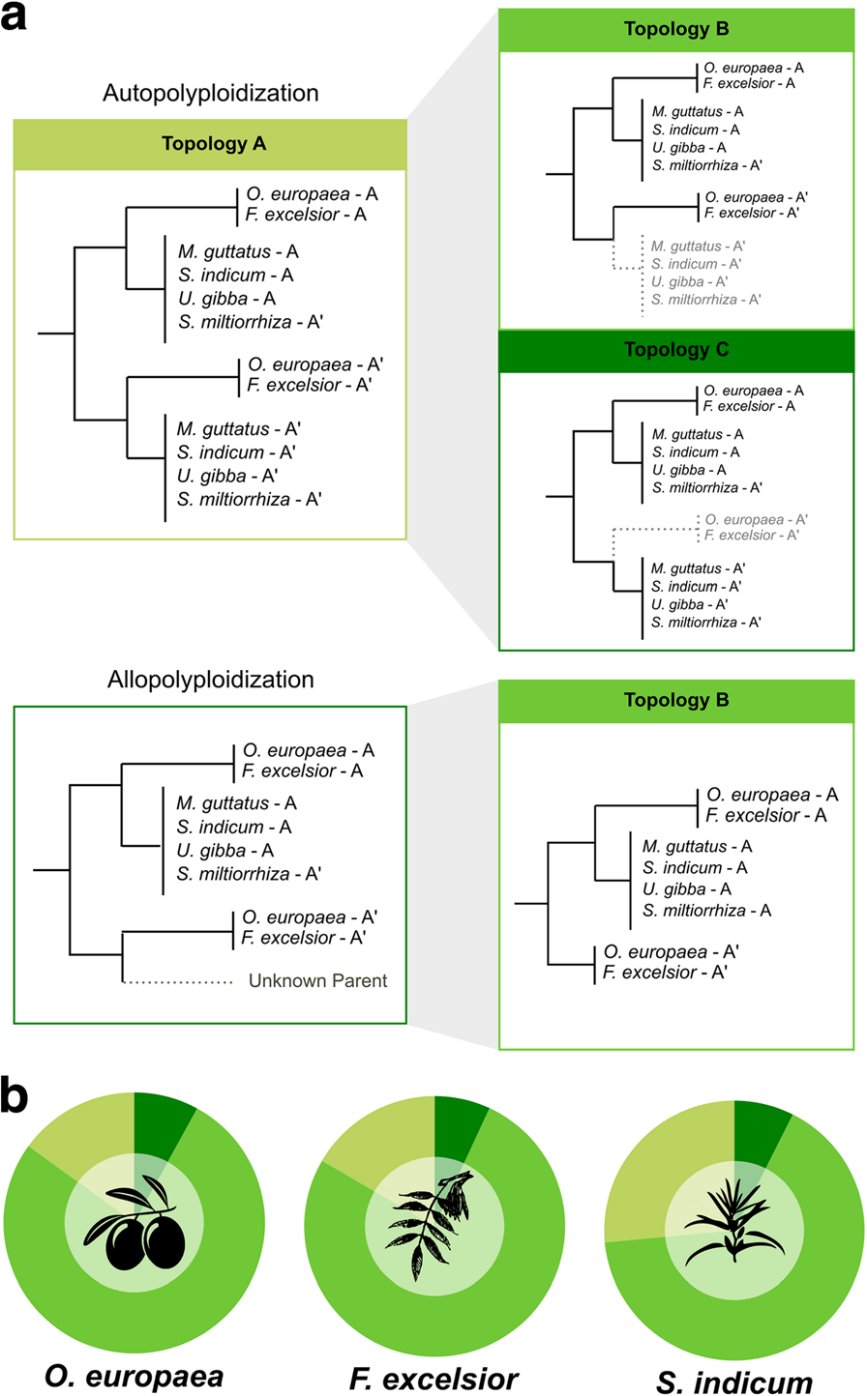
Topological analysis in olive and two other Lamiales. **a** Possible scenarios of duplication and loss, and resulting topologies. Top left: In an autopolyploidization scenario in the common ancestor of Oleaceae and the other non-Oleaceae Lamiales, topology A is produced, and would be expected in gene-loss scenarios where paralogous gene copies are maintained in at least one species from both Oleaceae and the non-Oleaceae Lamiales. Top right: Gene loss may produce topology B if one paralogous lineage is lost in all non-Oleaceae Lamiales species, or alternatively, topology C if one paralogous lineage is lost in all Oleaceae species. Alternatively, an allopolyploidization scenario in which one of the parental lineages is not sampled will directly result in topology B or topology C, depending on the phylogenetic position of such parental. An allopolyploidization scenario resulting in a preponderance of topology B is depicted here. **b** Percentage of gene trees that support each of the topologies shown in Fig. 2a in the phylomes of *O. europaea*, *F. excelsior*, and *S. indicum*

One unexplored aspect of the newly discovered WGD (node 4) was whether it was the result of an autopolyploidization or an allopolyploidization. To assess these two scenarios, we performed a topological analysis on the 10,670 gene trees in the olive phylome presenting duplications at this node (see “Methods”), and assessed how many supported each of three possible topologies (see Fig. 2a):

- Topology A: Both paralogous lineages maintain gene copies in at least one species from both Oleaceae and the non-Oleaceae Lamiales species*.*
- Topology B: One of the paralogous lineages was lost in all non-Oleaceae Lamiales species.
- Topology C: One paralogous lineage was lost in all Oleaceae species.

Our results showed a clear preponderance of topology B (Fig. 2b), with 77% of the trees in the *O. europaea* phylome supporting this topology. An equivalent analysis of the other Lamiales phylomes provided consistent results (see Fig. 2b and Additional file 4: Figure S3c).

The relative abundance of these three topologies can serve to distinguish between auto- and allopolyploidization. Indeed, autopolyploidization would initially result in topology A, with subsequent losses resulting in either topologies B or C (Fig. 2a). The more recent the autopolyploidization event and the lower the degree of gene loss, the higher the expected proportion of topology A in comparison with topologies B and C. In an autopolyploidization scenario, one would not expect notable differences between the abundance of topology B and topology C, assuming that both descendant clades are equally likely to lose a paralog. A clear preponderance of one of the loss topologies (i.e., topology B and topology C) is, however, expected from a hybridization scenario in which one of the parental lineages is not sampled. In our case, a preponderance of topology B, as we observe, could result from a hybridization event between an unsampled parental lineage with a lineage related to the non-Oleaceae Lamiales species included in our study (see Fig. 2a).

A preponderance of topology B is even less expected under an autopolyploidization scenario because it implies gene loss in the clade with more included species (four non-Oleaceae species vs. two Oleaceae species). If any, the effect of unbalanced taxon sampling should have been a preponderance of topology C and not topology B. We verified this by analyzing additional phylomes that contained a WGD event and unbalanced taxon sampling in the descendant lineages (Additional file 4: Figure S3b). Thus, our unbalanced taxon sampling in the lineages following the WGD cannot explain the observed preponderance of topology B, which is the expected one under a hybridization scenario. Altogether, our topological analyses support an allopolyploidization scenario for the duplication peak at node 4.

### Increased phylogenetic resolution provided by transcriptomes uncovers allopolyploidization at the base of the tribe Oleeae

The ability to discern the relative timing and type of polyploidizations depends on the taxonomic sampling of the compared genomes. Unfortunately, at the time of starting this analysis, the olive tree and *F. excelsior* were the only fully sequenced genomes from within the family Oleaceae. To increase the resolution of our analyses we included the transcriptomes of two Oleaceae species whose genomes are not available: *Jasminum sambac* [49] and *Phillyrea angustifolia* [50]. The two species plus *F. excelsior* represent three important divergence points in the olive lineage. *P. angustifolia* belongs to the same subtribe (Oleinae), *F. excelsior* belongs to the same tribe (Oleeae) and *J. sambac* belongs to the same family (Oleaceae). In addition, *J. sambac* has only 26 (2*n*) chromosomes, whereas the other three species have 46 chromosomes, which suggests that *J. sambac* likely experienced a lower number of polyploidizations. We, thus, expanded the olive phylome with these transcriptomes (see “Methods”). We then selected two sets of trees: namely those including at least one sequence of each newly included species (set 1: 20,705 trees) and those where a monophyletic clade contained the olive protein used as a seed in the phylogenetic reconstruction, and at least one sequence of each of the newly included species (set 2: 11,352).

**Fig. 3:**
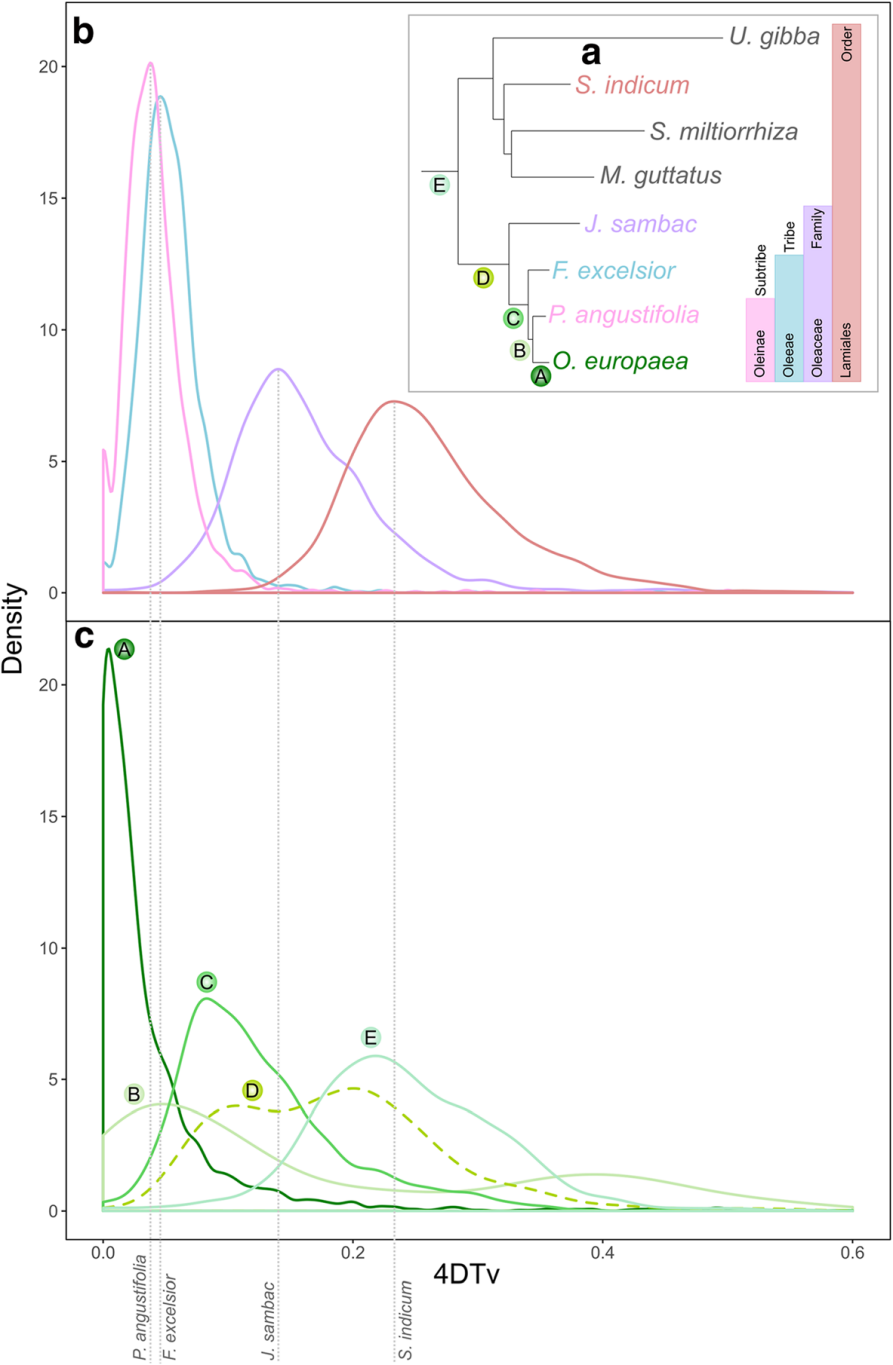
Species tree and 4DTv of set 1. **a** Species tree of the group of Lamiales including the additional two Oleaceae species. Bars on the right show the taxonomic classification. Duplication nodes for which 4DTv values of the paralogous pairs were calculated are marked with letters (A to E) as referred to in the text and colored according to each evolutionary age. The species used to calculate the 4DTv of orthologs pairs are shown in different colors. **b** Distributions of 4DTv values for the orthologous pairs between *O. europaea* and *P. angustifolia*, *F. excelsior*, *J. sambac*, and *S. indicum*, respectively. Colors correspond to the species in (**a**). The peaks of the orthologous pairs mark speciation events. Hence, considering that earlier events are on the left (lower 4DTv values), the order of speciations is in agreement with the species tree. **c** 4DTv of the paralogous pairs of *O. europaea* at the marked nodes in the tree. Each peak marks duplication events. Colors and letters correspond to nodes marked in (**a**)

Using the same approach described above, we reconstructed the phylogeny of the expanded set of species (Fig. 3a), which was congruent with previous analyses based on plastid DNA [51]. Additionally, we estimated their divergence times (see “Methods” and Additional file 5: Figure S4). The nodes in the new phylogeny were named from A to E (Fig. 3a), where E matched node 4 in the initial species tree (Fig. 1b). A new duplication profiling using set 1 suggests three main duplication peaks in Oleaceae at nodes A, C, and D (see Additional file 6: Figure S5). The node at the base of the family Oleaceae (node D) is of similar density as the peak found at the base of the Lamiales (node E), which we already described as an allopolyploidization event that happened at the base of the Oleaceae family. Another peak at the base of the Oleeae tribe (node C) is higher than the previous two peaks, as could be expected of a more recent event. A third peak (node A) was still found specifically in *O. europaea*, indicating that this duplication occurred after the divergence with *P. angustifolia*. These peaks are still prominent when duplication ratios are based on the more stringent set 2 (see Additional file 6: Figure S5).

To obtain an independent assessment of the relative age of duplications, we plotted the ratio of transversions at fourfold degenerate sites (4DTv) for pairs of paralogs mapped at each of the branches in Fig. 3a, and compared these ratios with those of orthologous pairs found between *O. europaea* and the three other Oleaceae species plus *S. indicum* (see Fig. 3 and Additional file 7: Figure S6). The resulting patterns (Fig. 3) indicate the overall congruence between topological dating and sequence divergence. The most recent duplication peak comprised olive-specific duplications and followed the separation of olive and *P. angustifolia* ~10 MyA (see Additional file 5: Figure S4). A second wave of duplications appeared after the divergence of *J. sambac* and before the divergence of *F. excelsior*, at the base of the Oleeae tribe, which diverged between 14 and 33 MyA. Interestingly, duplications that appeared in this region of the 4DTv correspond to duplications mapped to two different branches, according to our gene tree topological analyses: duplications at node C after the divergence of *J. sambac* and a fraction of the duplications mapped at node C preceding the divergence of *J. sambac*. The most ancient duplication wave corresponds to the allopolyploidization event that we have previously described, which occurred 33–72 MyA at the base of the Oleaceae family (node E). Of note, this time frame includes the Cretaceous–Tertiary (KT) mass extinction event, around which many other plant polyploidization events have been predicted [11]. That duplications whose topology map at node E are found in this region of the 4DTv, placed after the divergence of *S. indicum*, further supports the hybridization claim we first proposed using the topological analysis. Indeed, incongruence between inferred duplication ages and the time when the polyploidization has occurred is a clear indication of the presence of hybridization [28]. We also note that some of the duplications that map at node D are found in this region.

Altogether, these results confirm the presence of three waves of duplications but also show that the duplications that map at node D are divided into two peaks of sequence divergence, as indicated by 4DTv plots. Node D duplications with 4DTv values found between the divergence of *S. indicum* and *J. sambac* can be explained as a result of the proposed allopolyploidization at the base of Oleaceae, either by the loss of non-Oleaceae Lamiales species or by recombination where the non-Oleaceae Lamiales copy was overwritten (Additional file 8: Figure S7). The other fraction of node D duplications with 4DTv values that map after the speciation of *J. sambac* are more difficult to explain, as in the trees they predate *J. sambac* divergence. This scenario is similar to the one we observe at the base of Oleaceae (node E), where there is an incongruence between the relative age of duplicates estimated from sequence divergence and from gene tree topologies. Therefore, based on currently sequenced species, we propose that the tribe Oleeae was the result of a hybridization event with an ancestor in the lineage of *J. sambac* as one of the parents (Additional file 8: Figure S7). However, this conclusion may change in the future, as more genomes and transcriptomes become available. Still, our results support what Taylor proposed in 1945: that the Oleaceae group—with 23 chromosomes (Oleoideae)—had an allopolyploid origin whose ancestors were two (probably extinct) lineages from a group related to *Jasminum*, with chromosome numbers of 11 and 12 [52]. This scenario is further supported by the more stringent filtering of the trees (set 2). When at least one sequence of *J. sambac* is in the clade, then the duplication density at node D increases from 0.37 to 0.63 (Additional file 6: Figure S5). The use of a complete genome of *J. sambac* could further confirm this allopolyploidization hypothesis.

**Fig. 4:**
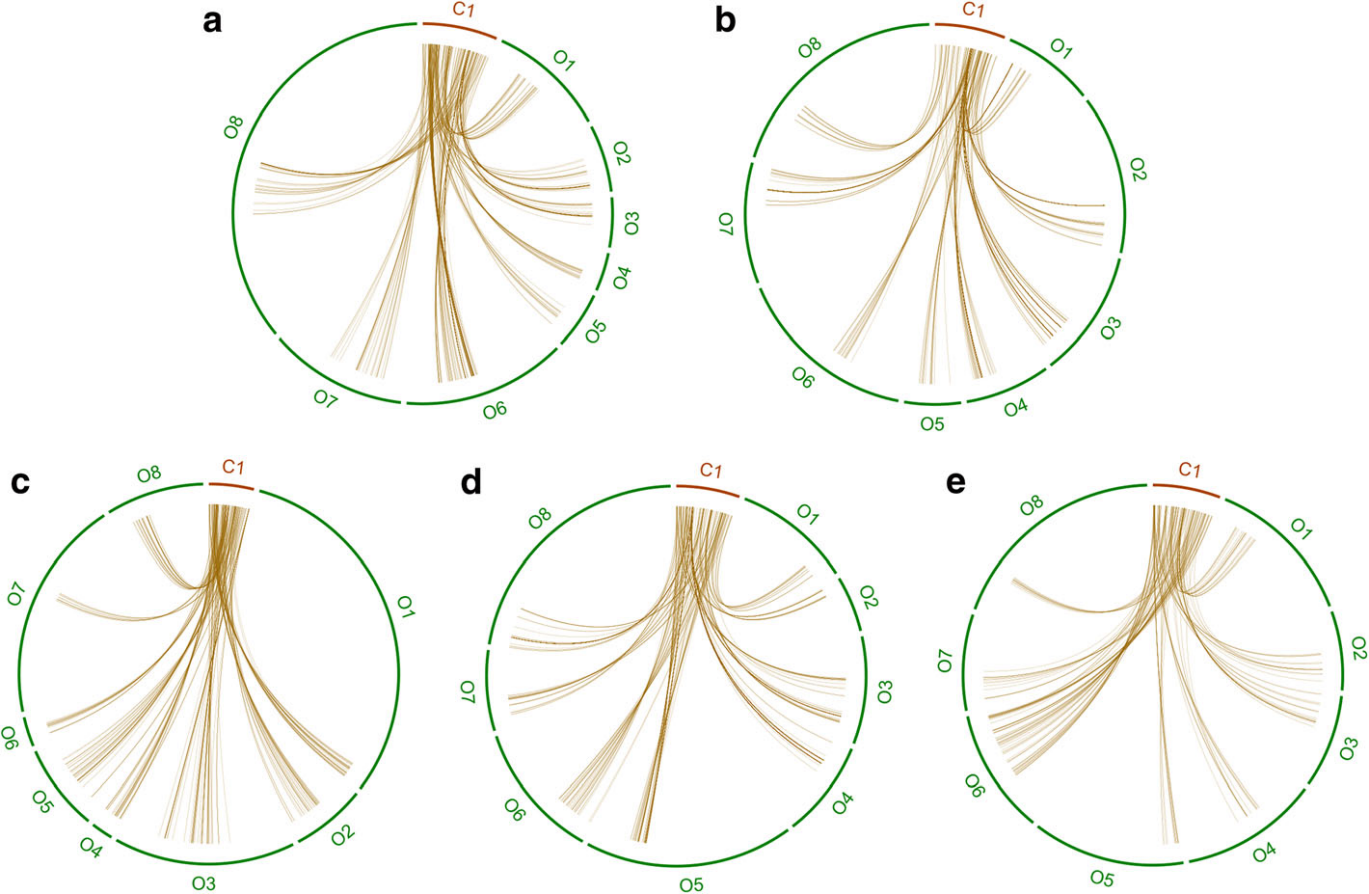
Example of five syntenic regions with a 1:8 relation between coffee ("C") and olive ("O"), as detected by GEvo. Exact regions corresponding to **a**, **b**, **c**, **d**, and **e** can be found in Additional file 2: Table S4

To confirm the two newly discovered allopolyploidization events with an alternative approach, we used GRAMPA [53], which relies on gene-tree and species-tree reconciliation to discern between allo- and autopolyploidization. We performed two different analyses. In the first, we compared the allopolyploidization model vs. the autopolyploidization model at the base of Lamiales (node E) (see Additional file 9: Figure S8a). We obtained lower parsimony scores for the allopolyploidization hypothesis (Additional file 2: Table S3), indicating a better match with the gene trees compared to an autopolyploidization scenario. We performed the same analysis comparing the proposed allopolyploidization at the base of the Oleeae lineage (node C) with two different hypotheses that place an autopolyploidization at the base of the family Oleaceae and at the base of the tribe Oleeae, respectively (see Additional file 9: Figure S8b). The results once again supported allopolyploidization over each of the two autopolyploidization hypotheses. Finally, inspection of the phylome identified examples of gene trees that retained the duplications of the three polyploidization events, and whose topology is congruent with the proposed scenario (see Additional file 10: Figure S9 as an example). Re-analysis of the syntenic depth results uncovered over 800 homologous syntenic regions with a depth of 8 between coffee and olive (see Fig. 4 and Additional file 2: Table S4).

### Comparison between the cultivated and wild Mediterranean *O. europaea* reinforces the possibility of a third polyploidization event

While this manuscript was under revision, another research group published the genome sequence of a wild Mediterranean olive tree or oleaster (*O. europaea* subsp. *europaea* var. *sylvestris*) from the eastern Mediterranean [37]. We used this opportunity to assess whether the most recent, cultivated olive-specific duplication is shared with oleaster. For this, we first reconstructed a phylome including both olive genomes and added the transcriptomes of *P. angustifolia* and *J. sambac* (see “Methods”). In the analysis of this new phylome, we selected two sets of gene trees as described before: set 1 (trees that include at least one sequence of each transcriptome) and set 2 (trees with a monophyletic clade containing the cultivated olive, oleaster, *P. angustifolia*, and *J. sambac*). As seen in Additional file 11: Figure S10, the duplication density is relatively high at the base of the two *O. europaea* genomes (0.28 for set 1 and 0.25 for set 2). This is in stark contrast with the previous node (ancestral of *P. angustifolia* and the olive), where a value of 0.03 indicates a lack of duplications at that branch. These results are supported by the 4DTv analysis, which shows that duplications that are mapped at the point of divergence between the two *O. europaea* genomes have a 4DTv density that falls before divergence of both olive trees, as marked by their ortholog divergence (see Additional file 12: Figure S11b). This result indicates that the most recent duplication wave occurred before the divergence of cultivated olive and oleaster and, hence, must have predated the domestication of the species. This is confirmed when using the number of synonymous substitutions per synonymous site (KS) values predicted by Synmap when comparing the two *O. europaea* genomes. The KS graph provided by Synmap presents five peaks (See Additional file 12: Figure S11c). The first is formed by proteins that were identical between both genomes. The last peak indicates mismatches when finding syntenic pairs. That leaves three peaks. To interpret correctly which genes formed these peaks, we checked whether the pairs of syntenic genes were orthologs or paralogs and if they were paralogs, at which point in the species tree they are duplicated. This shows that the difference in KS values between orthologs and paralogs that were duplicated during the WGD common to both olive genomes is so similar that the signal overlaps, through when represented separately, the peak of the orthologs is younger than that of the paralogs (Additional file 12: Figure S11d). The other two peaks correspond to the other two polyploidization events described before.

We note two puzzling features of this proposed olive-specific duplication. Firstly, the number of chromosomes in *Olea* is the same as that in *Fraxinus*, despite a putative specific duplication event in the former. This suggests that if the peak of duplicated genes results from a polyploidization event, then a return to the previous chromosome number must have happen relatively fast. Indeed, a rapid reduction of chromosome numbers has been observed in other families (i.e., within Brassicaceae [21]), which makes this scenario plausible. In contrast to chromosome numbers, several genome size parameters show differences between *Olea* and *Fraxinus*. For instance, experimentally inferred 1C genome sizes in picograms are higher in *Olea* than in *Fraxinus* according to the Plant DNA C-values database [54], and sequencing-based estimates of genome size of olive (1.32 Gb for the cultivated olive and 1.48 Gb for oleaster) [36, 37] are larger than that of *F. excelsior* (866.8 Mb) [38], as is the number of predicted proteins—56,349 for the cultivated olive and 50,684 for oleaster vs. 38,852 in *F. excelsior*.

A second puzzling observation is that the duplication density of around 0.25 (i.e., 25% of the genes duplicated after the divergence with *Fraxinus*) seems low for such a recent polyploidization. One possibility is that after so many polyploidization events, a large part of the genome was lost quickly due to the already existing redundancy, which would be compatible with a rapid return to a lower chromosomal number. Alternatively, the peak could be caused by numerous segmental duplications, uncoupled to a duplication in chromosome number. To assess that possibility, we analyzed the localization of paralogous genes and observed that they are not specific to a single region of the genome but are rather spread out over most scaffolds. From all the scaffolds that have at least one protein, 66.9% of scaffolds have at least one of the proteins that are duplicated. Also, 92.2% of the duplicated proteins have their paralogous pair in a different scaffold. These results indicate that the last duplication peak is indeed the result of a large-scale event covering most of the genomic regions, which strongly suggests a WGD scenario. Lastly, there is the possibility that the polyploidization event is so recent that many regions have not diverged sufficiently, resulting in many duplicated regions being collapsed during the assembly process. We explored this last possibility by comparing the two independent *O. europaea* genomes. The hypothesis is that the two independent assemblies may have collapsed different parts of the genome, due to different sequencing and assembly strategies, as well as different mutations being accumulated after the duplication. Our analyses of the phylome containing the two olive tree genomes support this idea. Out of the 4418 trees that have a well-supported duplication (aLRT (approximate Likelihood-Ratio Test) > 0.95) preceding the divergence of the two olive trees, only 770 (17%) show a topology where both olive genomes have retained the two copies derived from the duplication. Of these, 2962 (67%) show that only the cultivated olive retains the two paralogs, while in 686 (16 %) trees, the two paralogs are retained only by oleaster. This could indicate that the oleaster genome is more collapsed than the cultivated olive genome, which would be consistent with the fact that the assembly of only the cultivated variety used fosmid libraries and thus, the assembly started from larger contiguous regions [36]. Alternatively, or in addition, differential gene loss following the duplication could also account for the observed differences in the retention of paralogs.

To confirm the possibility of partial collapsing of duplicated regions in the assembly, we resorted to analyzing raw sequencing reads, which are available to us only for the cultivated variety [36]. We mapped such sequencing reads to the cultivated olive tree genome and identified heterozygous single-nucleotide polymorphisms (see “Methods”). Collapsed regions can be revealed by plotting the relative coverage of alternative alleles in heterozygous sites, as they may show an apparent higher ploidy. In general, for diploid organisms we should observe a single peak at 0.5 as two alleles should be present with identical frequency, a triploid should have two peaks near 0.33 and 0.66, and a tetraploid should have three peaks close to 0.25, 0.50, and 0.75 (See Additional file 13: Figure S12). As expected by the collapsed assembly hypothesis, we observed that many scaffolds did show a partial tetraploid pattern. We compared the relative coverage of alternative alleles in selected sets of duplicated genes showing the three possible topologies explained above (Additional file 14: Figure S13a):

- T1: A complete gene tree, meaning that both paralogous lineages conserve the proteins of cultivated olive and oleaster.
- T2: An incomplete gene tree, where one side lost the oleaster protein.
- T3: An incomplete gene tree, where one side lost the protein of the cultivated olive.

As expected under the assumption of a collapsed assembly, genes with topology T3 show the strongest tetraploid pattern compared to T1 and T2 (Additional file 14: Figure S13b). Altogether, these results indicate that both genome assemblies contain collapsed duplicated regions to a certain degree, which reduces the number of detected duplications in the olive-specific duplication peak.

## Conclusions

**Fig. 5:**
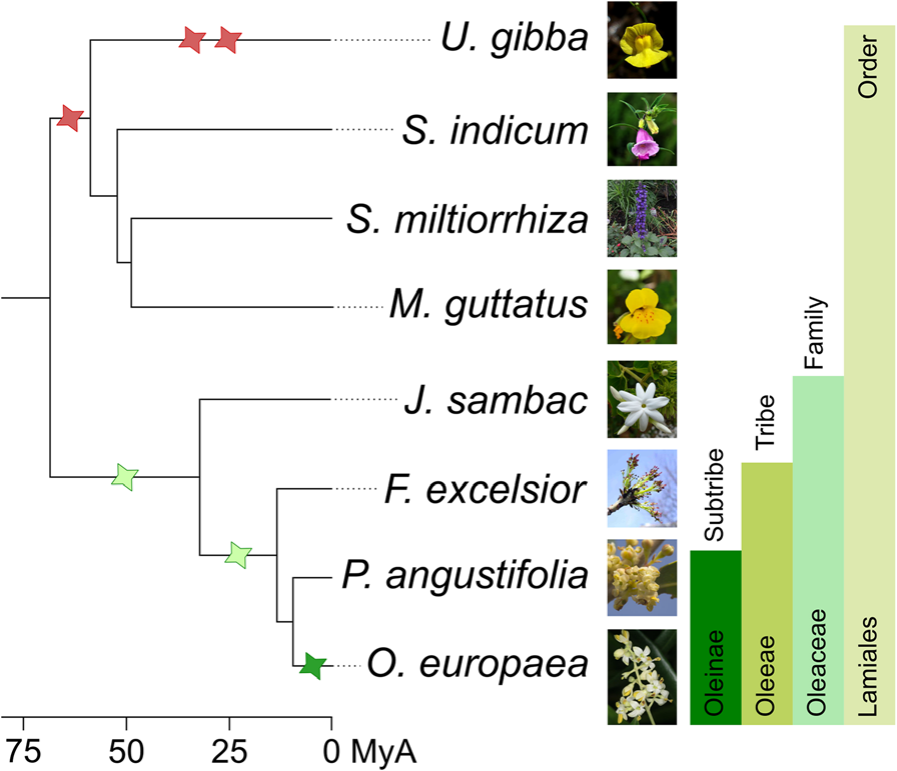
Species tree of the Lamiales clade showing the polyploidization events. Whole-genome duplications described in the literature are marked with red stars and whole-genome duplications described in this analysis are marked with green stars. The light green stars mark allopolyploidization events. Bars on the right show the taxonomic classification and the line at the bottom shows the divergence time in MyA

Altogether our results underscore the power of phylogenomics to distinguish between allo- and autopolyploidization. All our results indicate that the evolutionary history of the olive tree comprises not only a species-specific WGD, but also two ancestral allopolyploidization events (Fig. 5). The most ancestral paleoploidization occurred at the base of the family Oleaceae, where a non-Oleaceae Lamiales species could be involved as one of the parental species. Also, this event is independent of that described before for the lineage of non-Oleaceae Lamiales species. The second paleoploidization at the base of the tribe Oleeae seems to involve a species related to *Jasminum* as one of the partners, although increased taxonomic sampling may reveal other alternative scenarios. The third (neopolyploidization) event is specific to *O. europaea* and seems to be partially blurred by the fact that some duplicated regions may appear collapsed in the currently available assemblies. Future assembly efforts should consider this aspect. With the current set of sequenced species, we do not find phylogenetic support for an allopolyploidization scenario in which two *Olea* species hybridized to generate the modern olive tree. However, increased taxonomic sampling may change this. Finally, that *Fraxinus* and *Olea* have the same number of chromosomes may indicate that the last duplication event specific to olive was rapidly followed by genome rearrangements and with a quick return to the previous chromosome numbers. However, considering the ancient divergence (more than 35 MyA) between the two subtribes (Fraxininae and Oleeae) [51], alternative hypotheses may be considered. Further analyses and additional fully sequenced genomes from genera of Oleaceae are certainly needed to clarify these events better.

## Methods

### Gene order analysis

The comparative genomic tools in the CoGe software package [39] (https://genomevolution.org/coge/) were used to analyze gene order in the genomes of olive and its relatives. First, Synmap was used to compare the olive genome against itself using the Syntenic Path Assembly option [55] and to remove scaffolds without conserved synteny (see Additional file 1: Figure S1). Then, we used SynFind to obtain the syntenic depth, which is the number of conserved syntenic regions between the query genome and a reference. We obtained this value for comparisons of the olive, *Fraxinus excelsior*, and *Sesamum indicum* using *Coffea canephora* as reference (see Additional file 1: Figure S1). SynFind was also used to find regions with a 1:8 relationship between coffee and olive (see Fig. 4 and Additional file 2: Table S4).

Finally, Synmap was also used to compare the two *Olea europaea* varieties. A KS analysis was performed to find the number of putative polyploidization events that are shared between the two genomes. To interpret the results correctly, the evolutionary relationship between the genes providing the KS values was obtained from the phylome. Additionally, only genes found in clusters of at least size 3 were kept to try and focus only on syntenic groups that had the same relationship for all their genes.

### Phylome reconstruction

Eight phylomes were reconstructed. In all cases, an appropriate set of species was selected (see Additional file 2: Table S1) and the PhylomeDB automated pipeline was used to reconstruct a tree starting from each gene encoded in each one of the seed genomes [42]. This pipeline proceeds as follows. First, a Smith–Waterman search is performed [56] and the resulting hits are filtered based on the e-value and the overlap between query and hit sequences (e-value threshold < 1 × 10^-5^ and overlap > 0.5). The filtered results are then aligned using three different methods (MUSCLE v3.8, MAFFT v6.814b, and KALIGN 2.04) used in forward and reverse orientation [57–60]. A consensus alignment is reconstructed from these alignments using M-coffee [61]. This consensus alignment is then trimmed twice, first using a consistency score (0.1667) and then using a gap threshold (0.1) as implemented in trimAl v1.4 [62]. The resulting filtered alignment is subsequently used to reconstruct phylogenetic trees. To choose the best evolutionary model fitting each protein family, neighbor joining trees are reconstructed using BIONJ and their likelihoods are calculated using seven evolutionary models (JTT, WAG, MtREV, VT, LG, Blosum62, and Dayhoff). The model best fitting the data according to the Akaike information criterion is then used to reconstruct a maximum likelihood tree with PhyML v3.1 [63]. All trees and alignments are stored and can be downloaded or browsed in PhylomeDB [44] (http://phylomedb.org) with the Phylome IDs 215–222.

### Incorporation of transcriptomic data in the olive phylome

Transcriptome data was downloaded from the sources indicated in their respective publications: *Jasminum sambac* [49] and *Phillyrea angustifolia* [50]. For *J. sambac*, where no protein prediction derived from the transcriptome was available, we obtained the longest open reading frame (ORF) for each transcript. Only ORFs with a length of 100 aa or longer were kept, resulting in 20,952 ORFs for *J. sambac*. Transcriptomic data was introduced into each tree of the olive phylome using the following pipeline. First, a similarity search using blastP was performed from the seed protein against a database that contained the two transcriptomes. The results were then filtered based on three thresholds: e-value < 1 × 10^-5^, overlap between query and hit had to be at least 0.3, and a sequence identity threshold > 40.0%. Hits that passed these filters were incorporated into the raw alignment of the phylome using MAFFT (v 7.222) (--add and --reorder options) [64]. Then trees were reconstructed using the resulting alignment and following the same procedure as described above. Once all trees were reconstructed, they were filtered to remove unreliably placed transcriptome sequences. Phylomes tend to be highly redundant, especially when the seed genome contains many duplications, as is the case for the olive genome. Therefore, the same transcriptomic sequence is likely inserted in many trees. For each inserted transcript, we checked whether the sister sequences of each inserted transcript overlapped. If such an overlap did not exist, the transcript was deemed unreliable and removed from the tree. This filtered set was then filtered once more to select trees that contained at least one transcript for each of the two new species (set 1). Finally, set 1 was filtered again to keep only trees that contained a monophyletic clade including all the Oleaceae species (set 2).

### Species tree reconstruction

A species tree was reconstructed using data from olive phylome 215. Each tree reconstructed for this phylome was first pruned so that species-specific duplications were deleted from the tree, keeping only one sequence as representative of the duplicated group. Once trees were pruned, only those trees that contained one sequence for each of the 19 species included in the phylome were selected and 215 such trees were found. The clean alignments used to reconstruct these trees were concatenated and a species tree was reconstructed using the model of amino acids substitution that LG implemented in PhyML v3.1 [63] with 100 bootstrap replicates. In addition, a second species tree was reconstructed using a super-tree approach with the tool duptree [65]. In this case, all trees in the olive phylome were used for the tree reconstruction. A third species tree was reconstructed after including the transcriptomic data in the olive phylome. From the initial set of genes chosen to reconstruct the first species tree, a subset was chosen to reconstruct the extended species tree. This subset included only genes that incorporated at least one of the two species with a transcriptome. This final tree was reconstructed using 112 gene alignments using the same methodology as described above. Additional to these trees, a species tree for each of the other phylomes was reconstructed using the fasttree software v.2.1 [66] and the tool duptree.

### Detection and mapping of orthologs and paralogs

Orthologs and paralogs were detected using the species overlap method [26] as implemented in ETE v3.0 [67]. Species-specific duplications (expansions) are duplications that map only to one species, in our case always the species from which the phylome was started. To reduce the redundancy in the prediction of species-specific expansions, clustering was performed in which expansions that overlap in more than 50% of their sequences are fused together.

Predicted duplication nodes are then mapped to the species tree under the assumption that the duplication happened at the common ancestor of all the species included in the node, as described by Huerta-Cepas and Gabaldón [47]. Duplication frequencies at each node in the species tree are calculated by dividing the number of duplications mapped to a given node in the species tree by all the trees that contain that node. In all cases, duplication frequencies are calculated by excluding trees that contained large species-specific expansions (expansions that contained more than five members).

### Gene ontology term enrichment

Gene ontology (GO) terms were assigned to the olive proteome using interproscan [68] and the annotation of orthologs from the PhylomeDB database [44]. Phylome annotations were transferred to the olive proteome using one-to-one and one-to-many orthologs. GO term enrichment of proteins duplicated at the different species-specific expansions and duplication peaks was calculated using FatiGO [69].

### Topological analysis

A topological analysis was performed using ETE v3.0 [67] to test whether a duplication event happened at the base of Lamiales and to determine which species were involved. We searched how many trees supported each of the following topologies: the complete topology where at least one Oleaceae and at least one other non-Oleaceae Lamiales are found at both sides of the duplication (topology A), a partial topology where all non-Oleaceae Lamiales species have been lost in one side of the duplication (topology B), and another partial topology where the Oleaceae sequences have been lost at one side of the duplication (topology C) (see Fig. 2a). The analysis was then repeated for different previously reconstructed phylomes that contained ancient WGDs where there was an imbalance of species at either side of the duplication. The phylomes selected were those of the plants *Phaseolus vulgaris* [27] (Phylome ID 8) and *Solanum commersonii * [70] (Phylome ID 147), the fish *Scophthalmus maximus* [71] (Phylome ID 18), and the fungus *Rhizopus delemar* [24] (Phylome ID 252). Each of those phylomes contains an old WGD where at one side of the duplication there are less species than at the other one. We checked the proportion of trees that supported each topology. As with the Oleaceae example, topology A conserves at least one member of each group, topology B has lost all the species of the large group (set species 2) at one side of the duplication while topology C has lost all the species of the small group (set species 1) at one side of the duplication (see Additional file 4: Figure S3a).

We used GRAMPA [53] (spring 2016 version) to assess five different hypotheses (see Additional file 9: Figure S8) using the two sets of trees that contained transcriptomic data. This tool uses reconciliation to compute the support between a set of trees and a proposed allopolyploidization or autopolyploidization event, though it is limited to detecting one single event at a time. During its calculation, GRAMPA discards single gene trees that have too many possibilities when reconciling them to the species tree. The trees discarded can vary depending on the species tree hypothesis. Therefore, to compare fairly the parsimony scores obtained, we recalculated them based on the trees used in all the hypotheses. We performed two different analyses. In the first, we compared the allopolyploidization model vs. the autopolyploidization at the base of Lamiales (see Additional file 9: Figure S8a). In the second, we compared the allopolyploidization that led to the Oleeae lineage with two different hypotheses that place an autopolyploidization at the base of the Oleaceae family and at the base of the Oleeae tribe, respectively (see Additional file 9: Figure S8b). The results are in Additional file 2: Table S3.

### Transversion rate at fourfold degenerate sites (4DTv)

The 4DTv distribution was used to estimate speciation and polyploidization events. To obtain the gene pairs, we used the species trees that included the transcriptomic data, obtained from phylomes 215 and 221. For the first species tree, we calculated the 4DTv values for the orthologous gene pairs between *O. europaea* with *J. sambac*, *F. excelsior*, *P. angustifolia*, and *S. indicum*. We also calculated the 4DTv values for each paralogous gene pair of olive that maps at each evolutionary age of this tree. For the second tree, obtained from phylome 221 plus the transcriptomic data, we filtered the gene trees that had expansions larger than five involving both olives. Then, we calculated the 4DTv values for the orthologous pairs between the cultivated olive and oleaster. Also, we calculated the 4DTv values for each paralogous pair at the branches A, C, and E as marked in Additional file 12: Figure S11a.

### Divergence times

Divergence times were calculated using r8s-PL 1.81 [72]. Four nodes were taken as calibration points. The divergence times of these nodes were obtained from the TimeTree database [73]: *Mimulus guttatus* and *Arabidopsis thaliana* (117 MYA), *Sesamum indicum* and *Solanum lycopersicum* (84 MYA), *Glycine max* and *Arabidopsis thaliana* (106 MYA), *Zea mays* and *Solanum lycopersicum* (160 MYA). Cross-validation was performed to choose the smoothing parameter.

### Relative coverage of alternative alleles in heterozygous sites

To assess the ploidy of the cultivated olive genome using the relative coverage of alternative alleles in heterozygous positions, we first mapped the sequenced reads of this genome against itself using BWA [74]. Single-nucleotide polymorphisms were identified with GATK HaplotypeCaller v3.5 [75], by setting ploidy level 2 and using thresholds for mapping quality (>40) and read depth of coverage (>20). To get the number of reads that map at each heterozygous position, we used the SAMtools mpileup tool [76]. The relative coverage of alternative alleles was obtained by dividing the alternative allelic depth by the total depth at that position. For a diploid genome, we would expect a single peak around 0.50 at biallelic positions; for a triploid two peaks, around 0.33 and 0.67; and for a tetraploid three peaks, around 0.25, 0.50, and 0.75 (see Additional file 13: Figure S12).

For the analysis of the whole genome, we used scaffolds longer than 100 kb. In addition, to assess different scenarios in the *O. europaea*-specific duplications, we also computed the relative coverage of alternative alleles for proteins duplicated in the common ancestor of both olives. We used the list of genes from three gene tree topologies: (A) a complete gene tree, where both sides conserve var. *europaea* and *sylvestris*, (B) one side lost the *europaea* copy, and (C) one side lost the *sylvestris* copy. In all the cases, we used the gene trees obtained from phylome 221 and with at least five heterozygous positions.

## Additional files

Additional file 1: Figure S1. Results obtained with the CoGe package. **a** Image of a mapping of *O. europaea* against itself as shown by Synmap. **b** Syntenic depth of *O. europaea* (dark blue line), *F. excelsior* (light blue line), and *S. indicum* (blue dotted line) as calculated by SynFind. In all the comparisons, *C. canephora* was used as reference. (TIFF 749 kb)

Additional file 2: Table S1. List of species included in the reconstruction of the eight phylomes used in this study. Columns indicate, in this order, the species code for each species, the species name, the source for the protein and the coding DNA sequences, and the phylome in which the species was used (*O. europaea* var. *europaea*-215, *F. excelsior*-216, *M. guttatus*-217, *S. indicum*-218, *U. gibba*-219, *S. miltiorrhiza*-220, *O. e.* var. *europaea*-221, and *O.e.* var. *sylvestris*-222). **Table S2.** List of the GO terms enriched in the expanded protein families and at each evolutionary period as described in Fig. 1b. The first column shows the GO term, the second, the term level, the third, the *p* value, and the fourth, the term name. **Table S3.** List of parsimony scores for each of the different hypothesis shown in Additional file 9: Figure S8; and considering the two sets of trees with EST data. Nodes are named as shown in Fig. 3. **Table S4.** Syntenic regions between coffee and olive used in Fig. 4. In the first column, we can see the letter of the graph. The second and sixth columns show the scaffold names used in the graph (names starting with “C” are for coffee and “O” are for olive). The third and seventh columns show the scaffold names of the genome in coffee and olive, respectively. The fourth and fifth columns show the start and end of the region in coffee. The eighth and ninth columns show the start and end of the syntenic region in olive. (XLS 698 kb)

Additional file 3: Figure S2. Heat map showing the percentage of orthologous proteins between all Lamiales species included in this analysis. The values in the table represent the percentage of proteins of each seed species (rows) that have orthologs in each of the other species (columns), as computed from the corresponding phylome. For instance, 53% of *F. excelsior* proteins have orthologs in *U. gibba*. Conversely, 82% of *U. gibba* proteins have orthologs in *F. excelsior*. (TIFF 501 kb)

Additional file 4: Figure S3. Topological analysis of seven different species. **a** Possible scenarios and alternative topologies after a duplication. Topology A (TA): After the duplication, both sides maintain gene copies in at least one species of set species 1 and set species 2. TB: One of the sides lost all of set species 2. TC: One of the sides lost all of set species 1. **b** Pie charts representing the distribution of gene trees supporting each of the different topologies for the phylomes of *Phaseolus vulgaris* (bean), *Solanum commersonii* (wild potato), *Scophthalmus maximus* (fishes), and *Rhizopus delemar* (Mucoromycotina), taken from PhylomeDB. **c** Percentage of gene trees supporting each topology for the phylomes of *U. gibba*, *S. miltiorrhiza*, and *M. guttatus.* (TIFF 854 kb)

Additional file 5: Figure S4. Chronogram depicting the evolution of the plants included in phylome 215 plus transcriptomes. Green dots represent selected calibration points in MyA. (TIFF 818 kb)

Additional file 6 **Figure S5**. Species tree of the order Lamiales, including *P. angustifolia* and *J. sambac*. The duplication rates are shown in red for set 1 (gene trees that included genes of *J. sambac* and *P. angustifolia*) and in blue for set 2 (gene trees that have a monophyletic clade of the family Oleaceae). The gray circles show the node name and the bars on the right, the taxonomic classification. (TIFF 1010 kb)

Additional file 7: Figure S6. Species tree and 4DTv of set 2. **a** Species tree of the group of Lamiales including the four Oleaceae species. Nodes where the 4DTv of the paralogous pairs were calculated are marked with letters (A to E) as referred to in the text and colored according to each evolutionary age. The species used to calculate the 4DTv of orthologous pairs are shown in different colors. The bars on the right show the taxonomic classification. **b** 4DTv of the orthologous pairs between *O. europaea* with *P. angustifolia*, *F. excelsior*, *J. sambac*, and *S. indicum*. **c** 4DTv of the paralogous pairs of *O. europaea* at the marked nodes in the species tree in (**a**). (TIFF 1049 kb)

Additional file 8: Figure S7. Schematic explanation of the 4DTv density at node D in Fig. 3c. **a** Representation of the two allopolyploidization events and the potential parentals. **b** A gene tree where the protein of *J. sambac* maps after the divergence of this species. **c** A gene tree where the non-Oleaceae Lamiales proteins are lost. **d** 4DTv of the paralogs at nodes C, D, and E. The dotted lines mark the divergence time between olive *J. Sambac* and olive *S. indicum*. (TIFF 744 kb)

Additional file 9: Figure S8. Phylogenetic trees representing the comparisons done for GRAMPA. In all cases, branches in green or orange represent the species that the polyploidy has affected. **a** The trees represent the hypothesis of an allopolyploidization vs. an autopolyploidization at the base of Lamiales. **b** These trees represent the hypothesis of an allopolyploidization at the base of the tribe Oleeae vs. two models of autopolyploidization (at the base of the family Oleaceae and at the base of the tribe Oleeae). (TIFF 1392 kb)

Additional file 10: Figure S9. Example gene tree that shows the three events we have described in olive: the species-specific duplication and the two allopolyploidizations. The whole-genome duplication previously described in non-Oleaceae Lamiales and the species-specific duplications in *U. gibba* can also be seen. (TIFF 1402 kb)

Additional file 11: Figure S10. Species tree of the family Oleaceae, including *P. angustifolia*, *F. excelsior*, *J. sambac*, *Olea europaea* subsp. *europaea* var. *europaea*, and *Olea europaea* subsp. *europaea* var. *sylvestris*. The duplication rates are shown in red for set 1 (gene trees that included genes of *J. sambac* and *P. angustifolia*) and in blue for set 2 (gene trees that have a monophyletic clade of the family Oleaceae). The bars on the right show the taxonomic classification. (TIFF 494 kb)

Additional file 12: Figure S11. 4DTv and KS including the two Mediterranean olives. **a** Species tree of the Lamiales order. Nodes where the 4DTv of the paralogous pairs were calculated are marked with letters (A, C, and E) and colored according each evolutionary age. The species used to calculate the 4DTv of orthologous pairs are shown in yellow. The bars on the right show the taxonomic classification. **b** 4DTv showing the orthologous between cultivated olive and oleaster (yellow), and the paralogous of each of the branches marked in the species tree in (**a**). **c** KS plot obtained from CoGe. **d** KS for genes found in syntenic regions with at least three pairs of genes that evolved at the same evolutionary time. (TIFF 703 kb)

Additional file 13: Figure S12. The relative coverage of alternative alleles in heterozygous sites. We assume that we have only two alleles. **a** In a diploid organism, for the heterozygous positions, we will have one option and, for instance, we will observe one single peak at 0.5. **b** In a triploid organism, we will have two options for the heterozygous positions (1/3 or 2/3), so in the plot we will observe two peaks at 0.33 and 0.67. **c** For a tetraploid organism, we will have three options (1/4, 2/4, and 3/4) so we will observe three peaks at 0.25, 0.50, and 0.75. (TIFF 264 kb)

Additional file 14: Figure S13. Relative coverage of alternative alleles in heterozygous sites of a tree with different lists of proteins. **a** Gene tree topologies used to get the olive proteins. T1: A complete gene tree, where both sides conserve both var. *europaea* and *sylvestris*. T2: One side of the gene tree has lost the *sylvestris* copy. T3: One side of the gene tree has lost the *europaea* copy. **b** Relative coverage of the alternative alleles in heterozygous sites for each of the gene lists as obtained from the tree gene topologies. (TIFF 493 kb)
