# Supplementary figures and images for: Phylogenomics of the olive tree (Olea europaea) reveals the relative contribution of ancient allo- and autopolyploidization events
Source: BMC Biol. 2018 Jan 25;16:15. doi: 10.1186/s12915-018-0482-y (PMC5785856; doi:10.1186/s12915-018-0482-y)

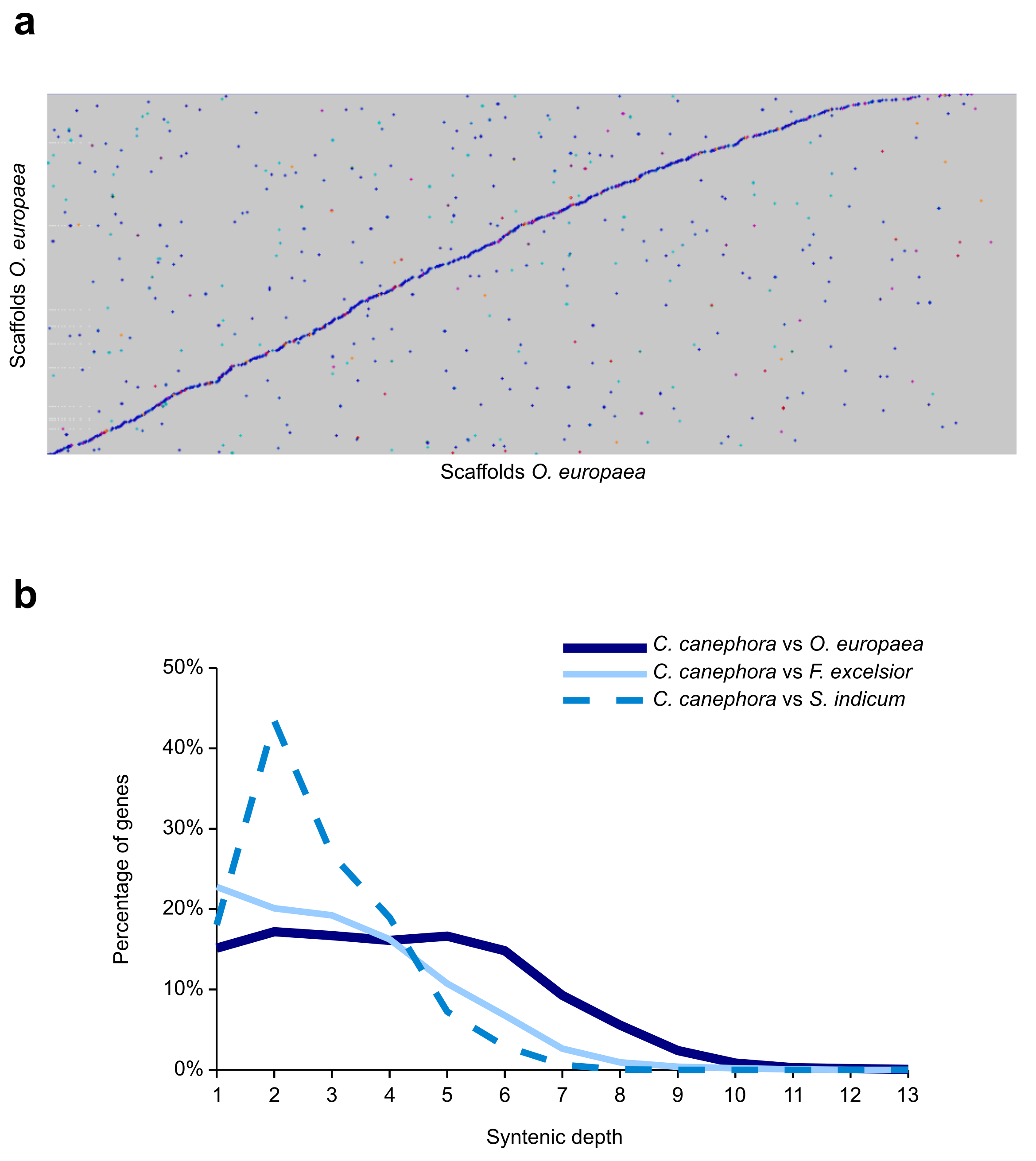

Supplement: Supplementary file 1 — Results obtained with the CoGe package. a Image of a mapping of O. europaea against itself as shown by Synmap. b Syntenic depth of O. europaea (dark blue line), F. excelsior (light blue line), and S. indicum (blue dotted line) as calculated by SynFind. In all the comparisons, C. canephora was used as reference. (TIFF 749 kb) [file 12915_2018_482_MOESM1_ESM.tiff]

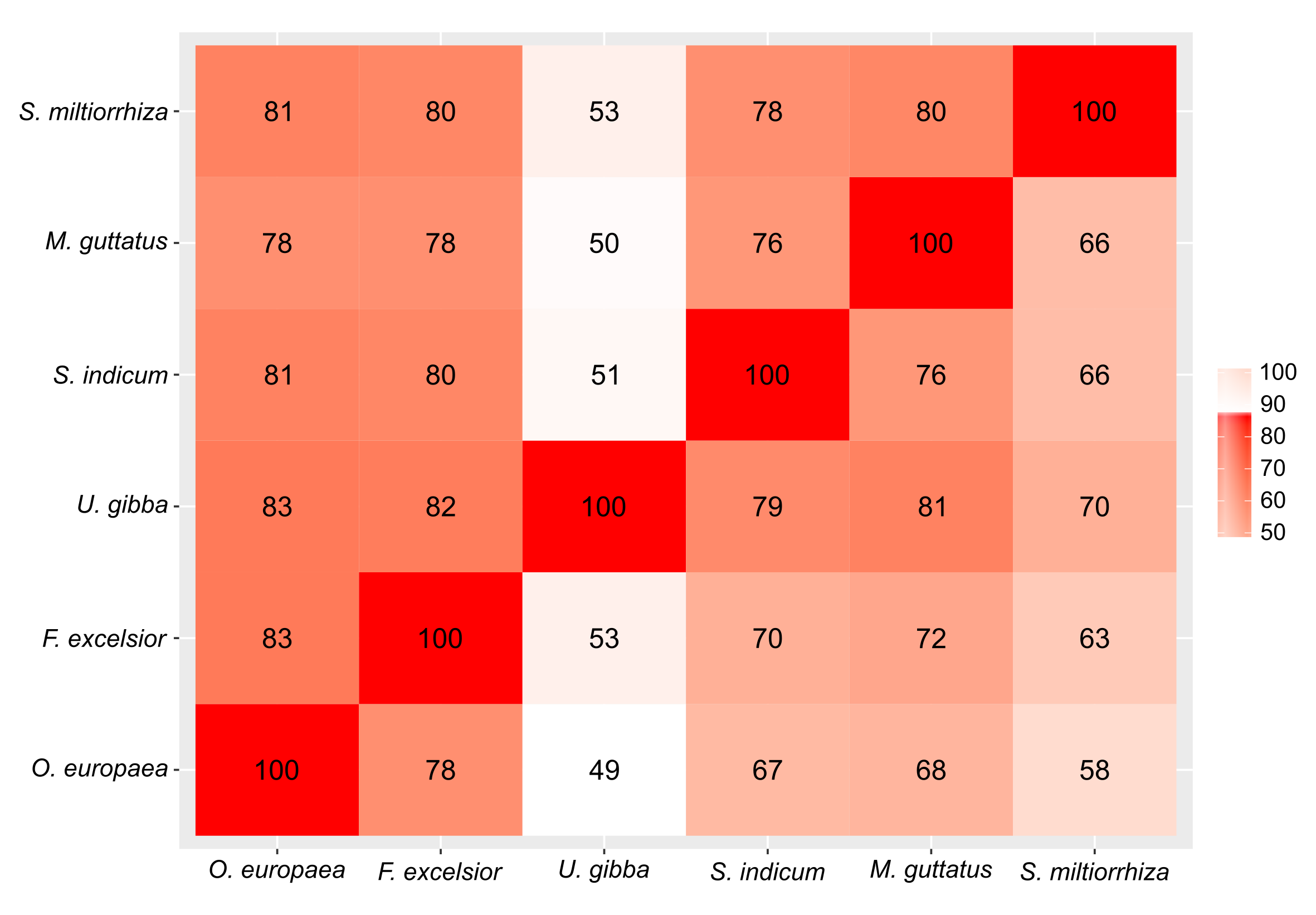

Supplement: Supplementary file 3 — Heat map showing the percentage of orthologous proteins between all Lamiales species included in this analysis. The values in the table represent the percentage of proteins of each seed species (rows) that have orthologs in each of the other species (columns), as computed from the corresponding phylome. For instance, 53% of F. excelsior proteins have orthologs in U. gibba. Conversely, 82% of U. gibba proteins have orthologs in F. excelsior. (TIFF 501 kb) [file 12915_2018_482_MOESM3_ESM.tiff]

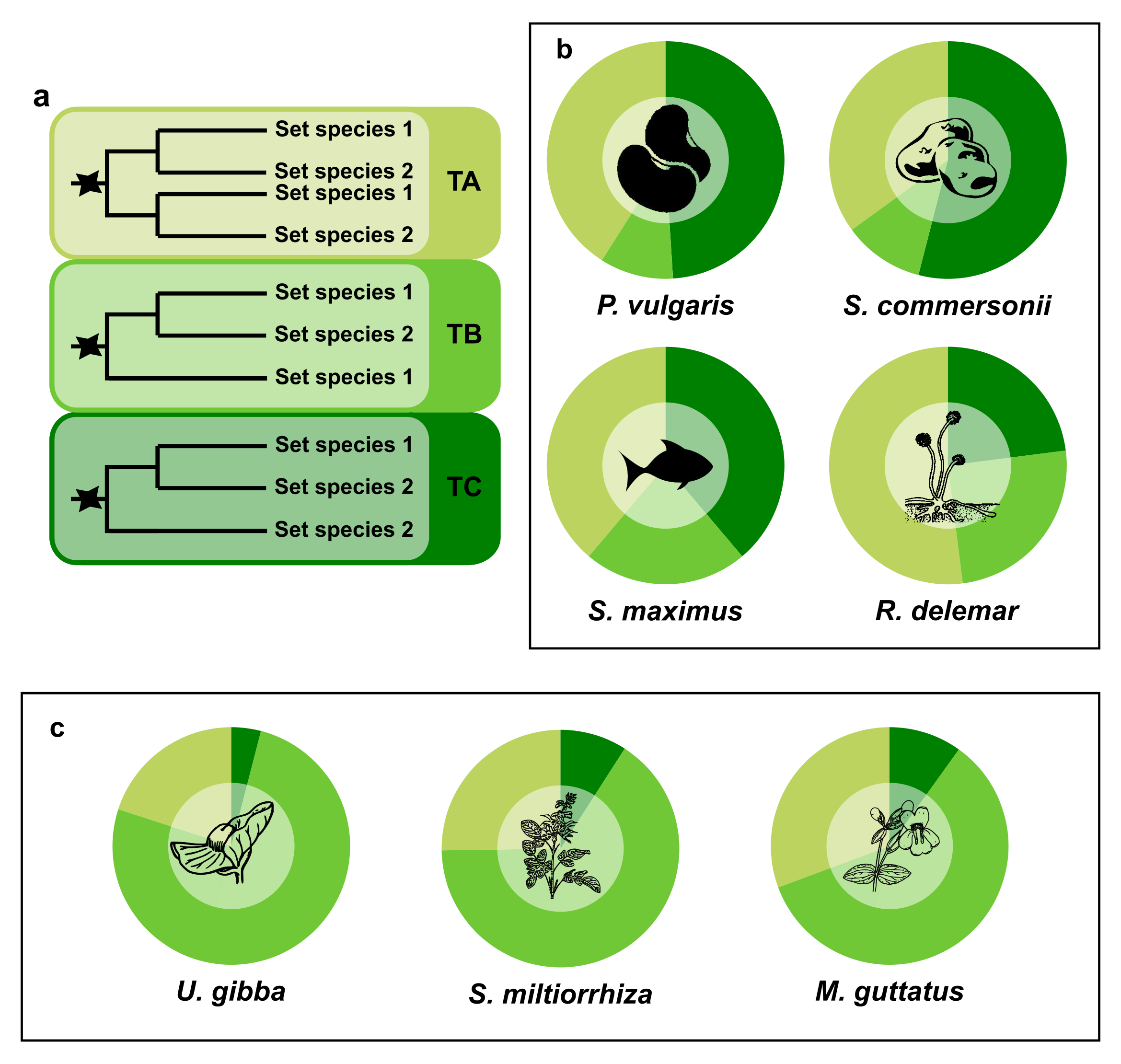

Supplement: Supplementary file 4 — Topological analysis of seven different species. a Possible scenarios and alternative topologies after a duplication. Topology A (TA): After the duplication, both sides maintain gene copies in at least one species of set species 1 and set species 2. TB: One of the sides lost all of set species 2. TC: One of the sides lost all of set species 1. b Pie charts representing the distribution of gene trees supporting each of the different topologies for the phylomes of Phaseolus vulgaris (bean), Solanum commersonii (wild potato), Scophthalmus maximus (fishes), and Rhizopus delemar (Mucoromycotina), taken from PhylomeDB. c Percentage of gene trees supporting each topology for the phylomes of U. gibba, S. miltiorrhiza, and M. guttatus. (TIFF 854 kb) [file 12915_2018_482_MOESM4_ESM.tiff]

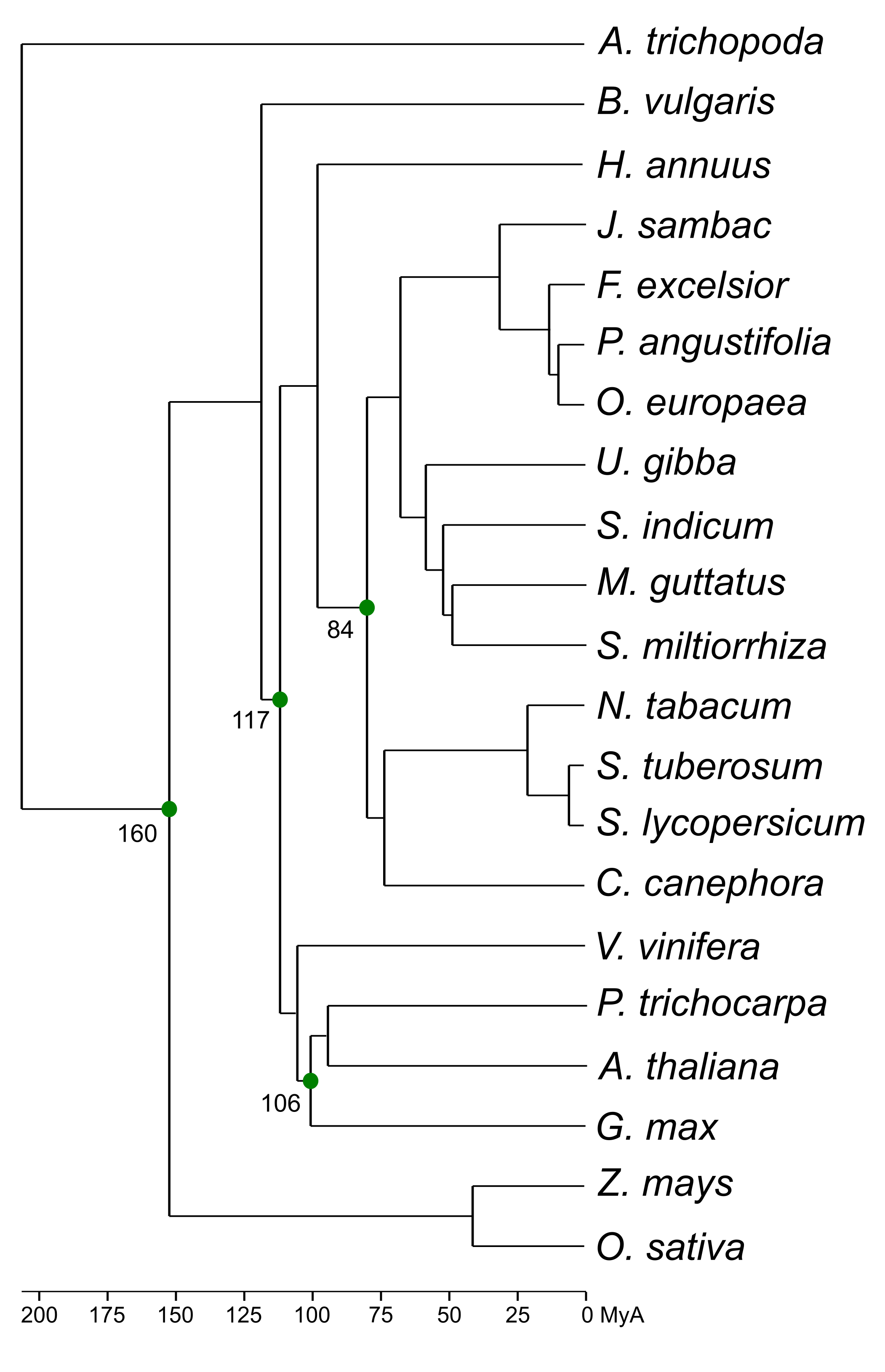

Supplement: Supplementary file 5 — Chronogram depicting the evolution of the plants included in phylome 215 plus transcriptomes. Green dots represent selected calibration points in MyA. (TIFF 818 kb) [file 12915_2018_482_MOESM5_ESM.tiff]

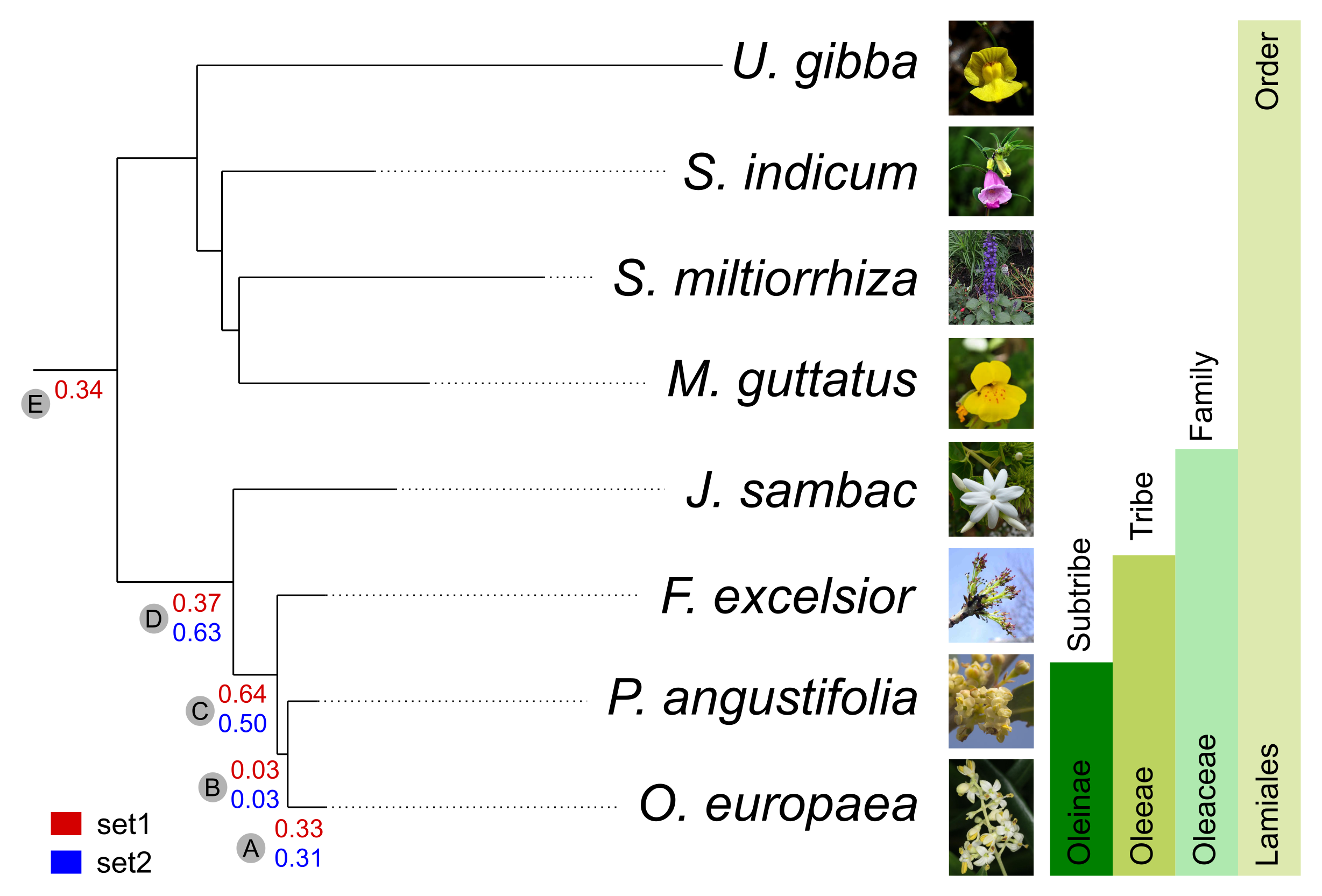

Supplement: Supplementary file 6 — Figure S5. Species tree of the order Lamiales, including P. angustifolia and J. sambac. The duplication rates are shown in red for set 1 (gene trees that included genes of J. sambac and P. angustifolia) and in blue for set 2 (gene trees that have a monophyletic clade of the family Oleaceae). The gray circles show the node name and the bars on the right, the taxonomic classification. (TIFF 1010 kb) [file 12915_2018_482_MOESM6_ESM.tiff]

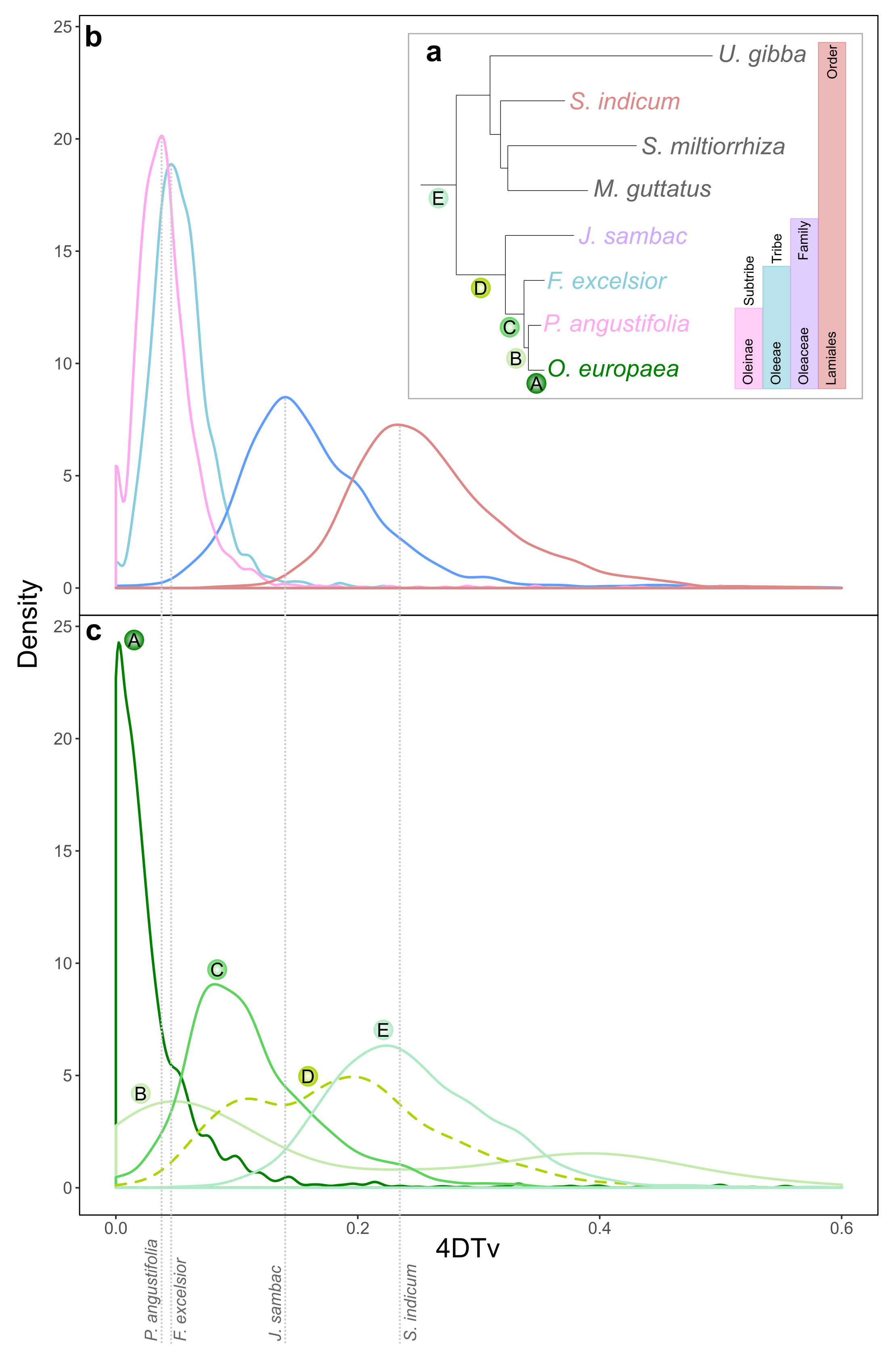

Supplement: Supplementary file 7 — Species tree and 4DTv of set 2. a Species tree of the group of Lamiales including the four Oleaceae species. Nodes where the 4DTv of the paralogous pairs were calculated are marked with letters (A to E) as referred to in the text and colored according to each evolutionary age. The species used to calculate the 4DTv of orthologous pairs are shown in different colors. The bars on the right show the taxonomic classification. b 4DTv of the orthologous pairs between O. europaea with P. angustifolia, F. excelsior, J. sambac, and S. indicum. c 4DTv of the paralogous pairs of O. europaea at the marked nodes in the species tree in (a). (TIFF 1049 kb) [file 12915_2018_482_MOESM7_ESM.tiff]

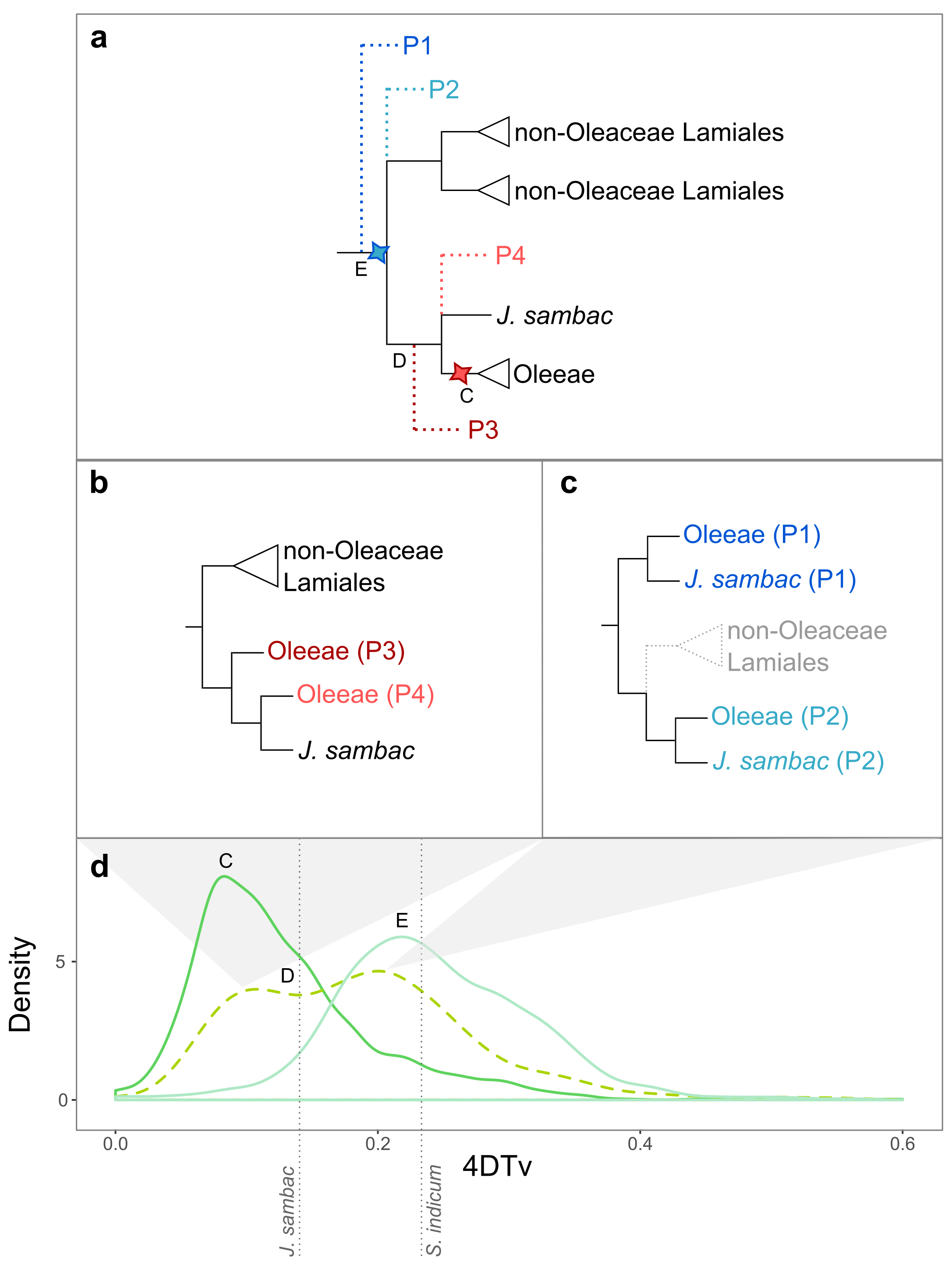

Supplement: Supplementary file 8 — Schematic explanation of the 4DTv density at node D in Fig. 3c. a Representation of the two allopolyploidization events and the potential parentals. b A gene tree where the protein of J. sambac maps after the divergence of this species. c A gene tree where the non-Oleaceae Lamiales proteins are lost. d 4DTv of the paralogs at nodes C, D, and E. The dotted lines mark the divergence time between olive J. Sambac and olive S. indicum. (TIFF 744 kb) [file 12915_2018_482_MOESM8_ESM.tiff]

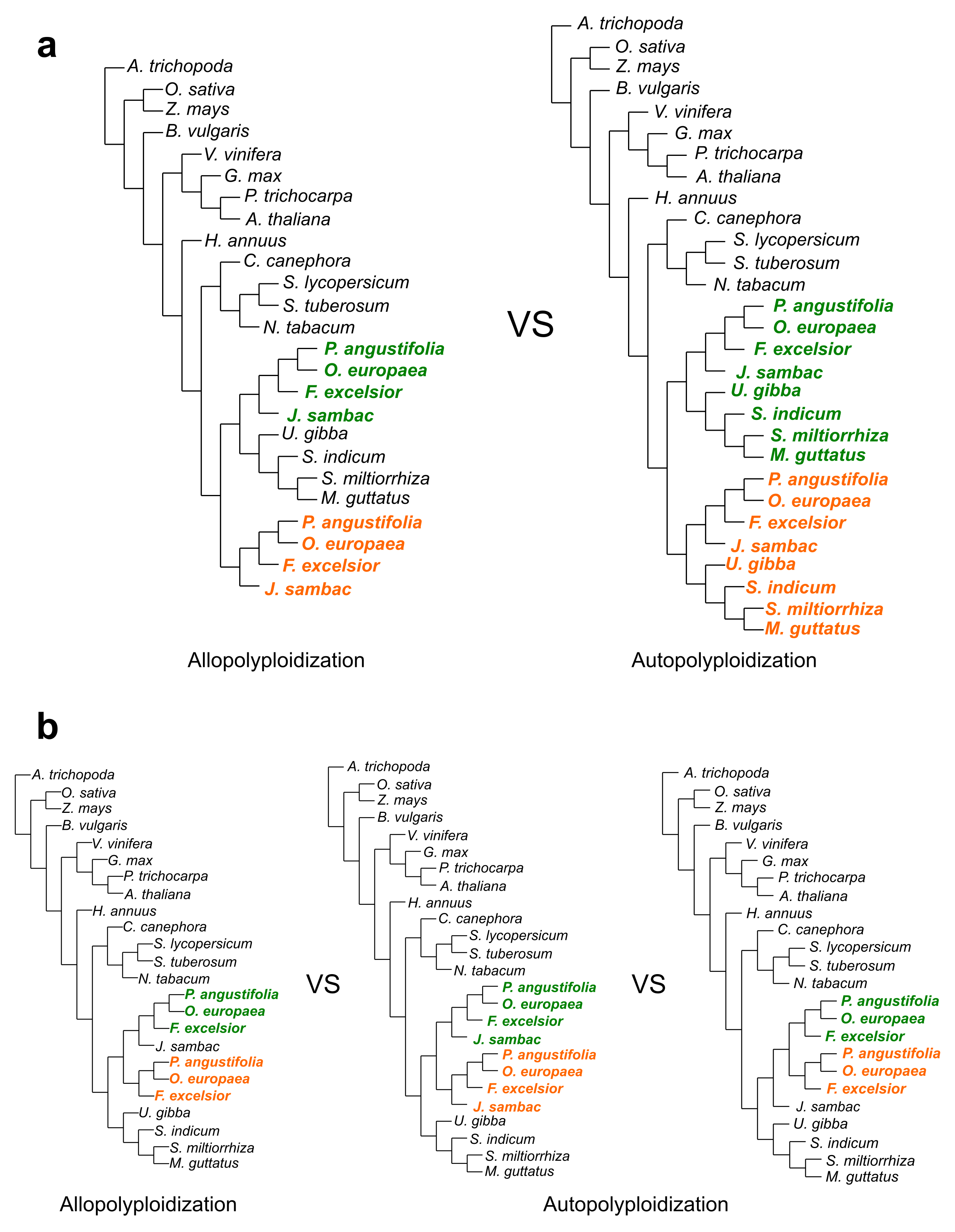

Supplement: Supplementary file 9 — Phylogenetic trees representing the comparisons done for GRAMPA. In all cases, branches in green or orange represent the species that the polyploidy has affected. a The trees represent the hypothesis of an allopolyploidization vs. an autopolyploidization at the base of Lamiales. b These trees represent the hypothesis of an allopolyploidization at the base of the tribe Oleeae vs. two models of autopolyploidization (at the base of the family Oleaceae and at the base of the tribe Oleeae). (TIFF 1392 kb) [file 12915_2018_482_MOESM9_ESM.tiff]

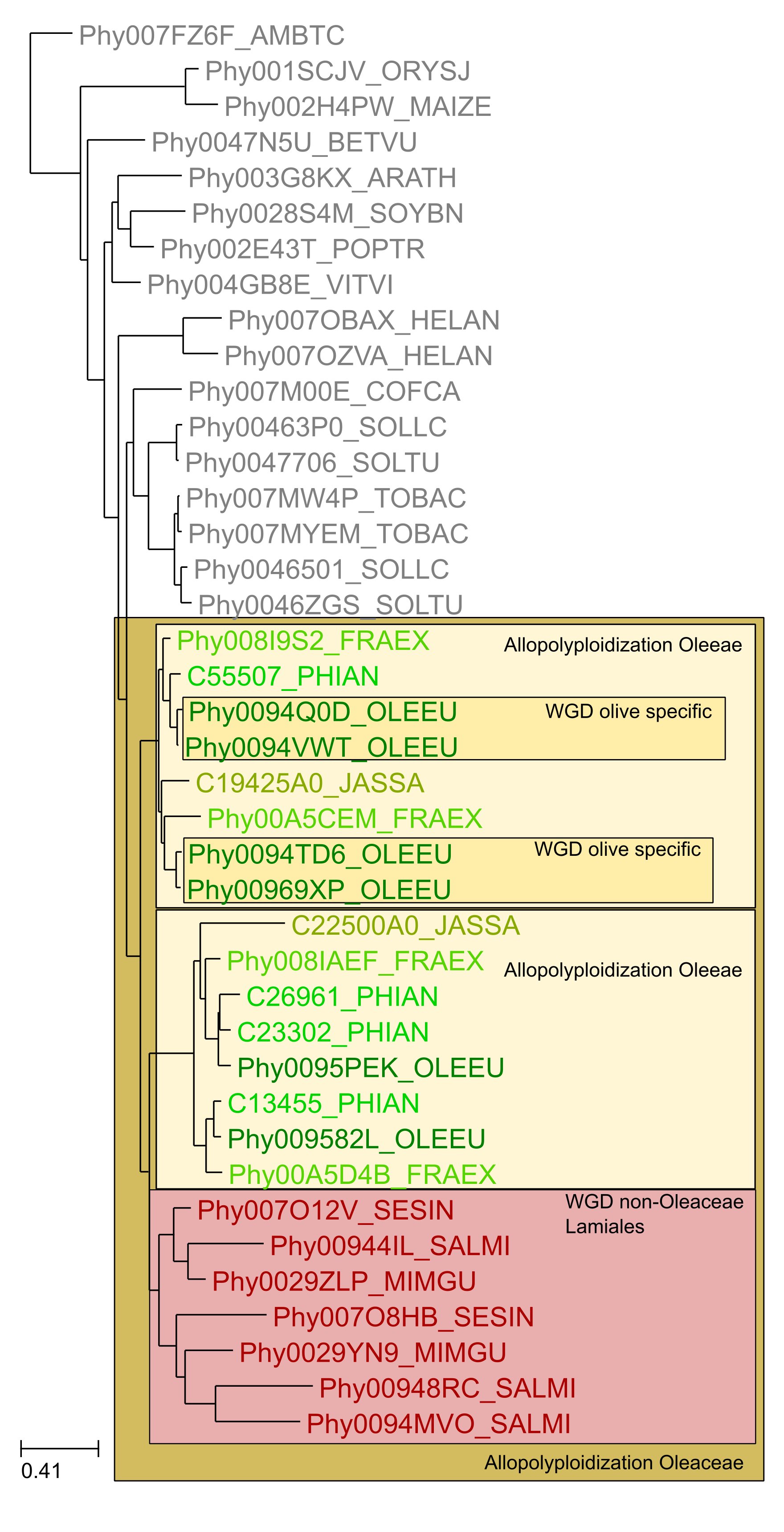

Supplement: Supplementary file 10 — Example gene tree that shows the three events we have described in olive: the species-specific duplication and the two allopolyploidizations. The whole-genome duplication previously described in non-Oleaceae Lamiales and the species-specific duplications in U. gibba can also be seen. (TIFF 1402 kb) [file 12915_2018_482_MOESM10_ESM.tiff]

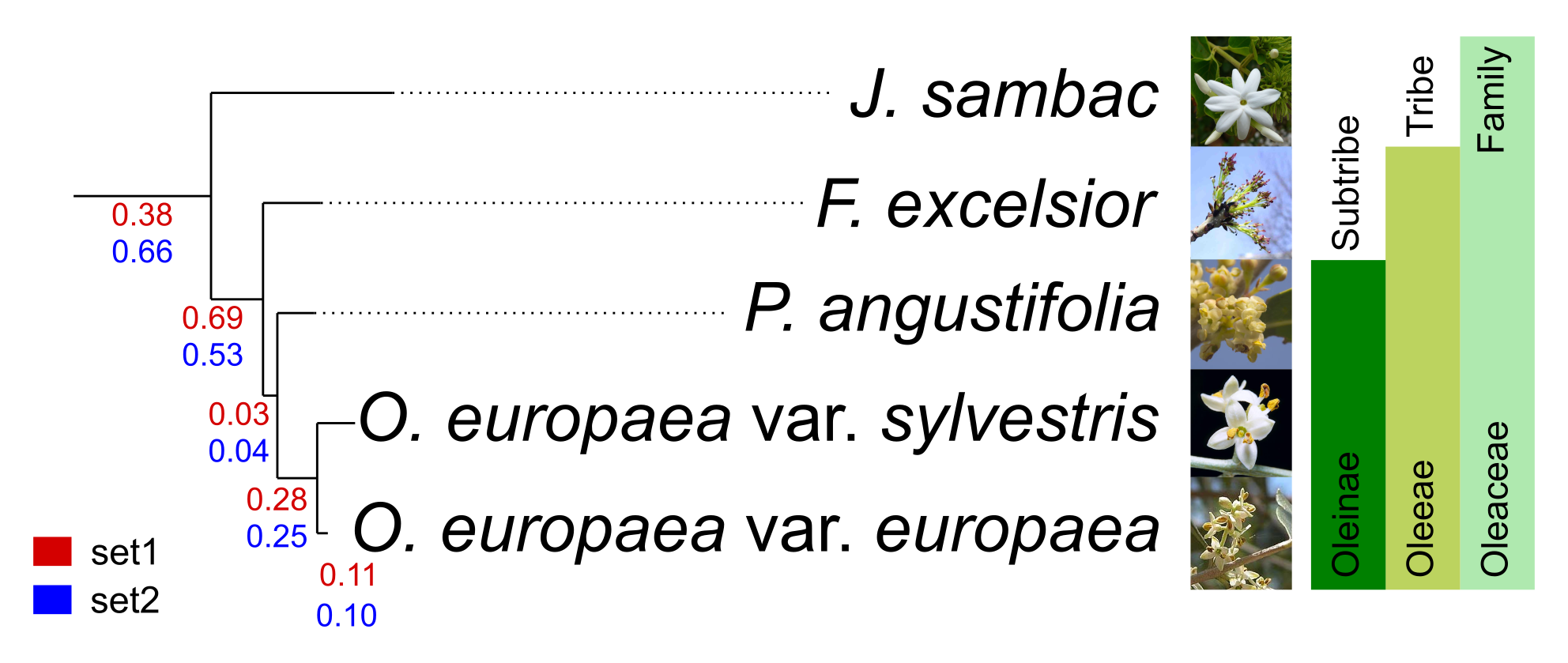

Supplement: Supplementary file 11 — Species tree of the family Oleaceae, including P. angustifolia, F. excelsior, J. sambac, Olea europaea subsp. europaea var. europaea, and Olea europaea subsp. europaea var. sylvestris. The duplication rates are shown in red for set 1 (gene trees that included genes of J. sambac and P. angustifolia) and in blue for set 2 (gene trees that have a monophyletic clade of the family Oleaceae). The bars on the right show the taxonomic classification. (TIFF 494 kb) [file 12915_2018_482_MOESM11_ESM.tiff]

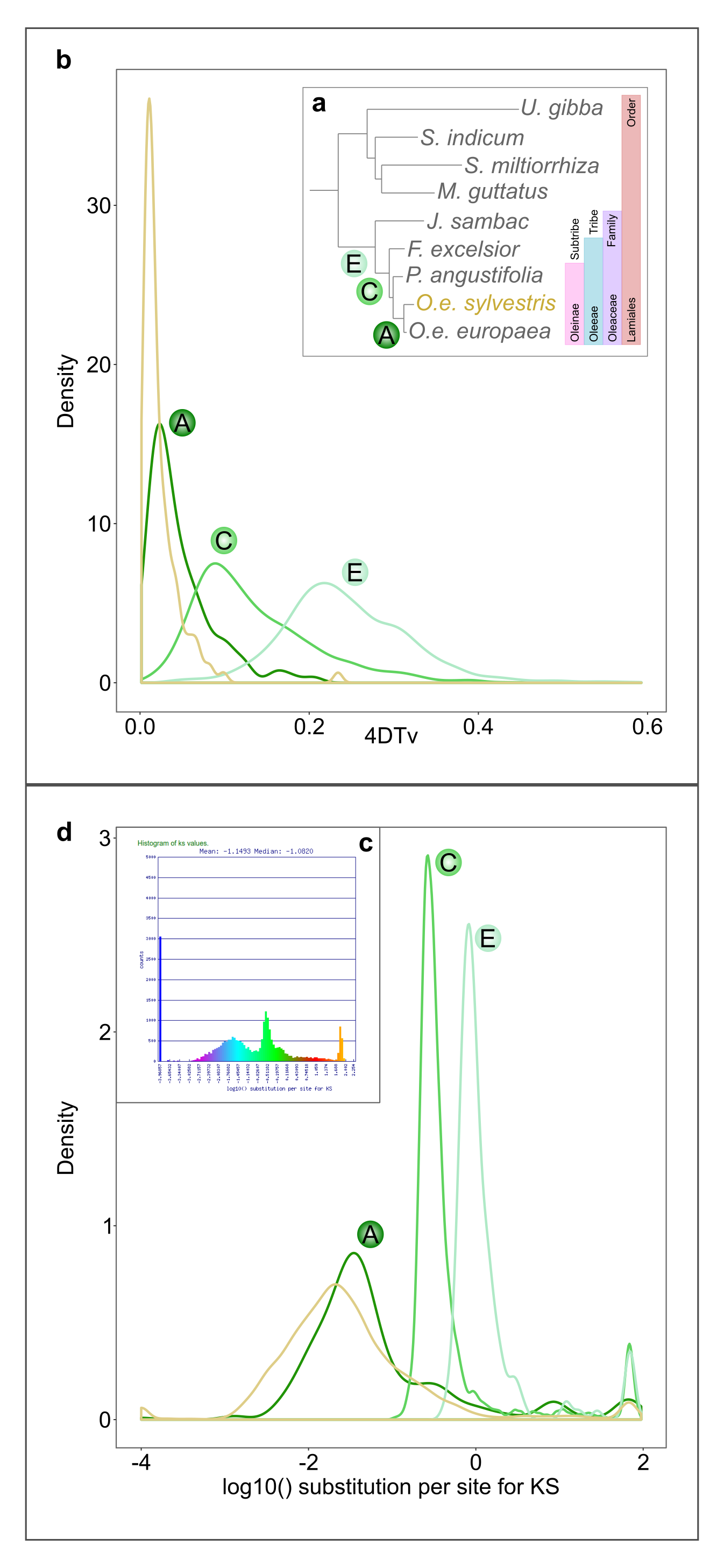

Supplement: Supplementary file 12 — 4DTv and KS including the two Mediterranean olives. a Species tree of the Lamiales order. Nodes where the 4DTv of the paralogous pairs were calculated are marked with letters (A, C, and E) and colored according each evolutionary age. The species used to calculate the 4DTv of orthologous pairs are shown in yellow. The bars on the right show the taxonomic classification. b 4DTv showing the orthologous between cultivated olive and oleaster (yellow), and the paralogous of each of the branches marked in the species tree in (a). c KS plot obtained from CoGe. d KS for genes found in syntenic regions with at least three pairs of genes that evolved at the same evolutionary time. (TIFF 703 kb) [file 12915_2018_482_MOESM12_ESM.tiff]

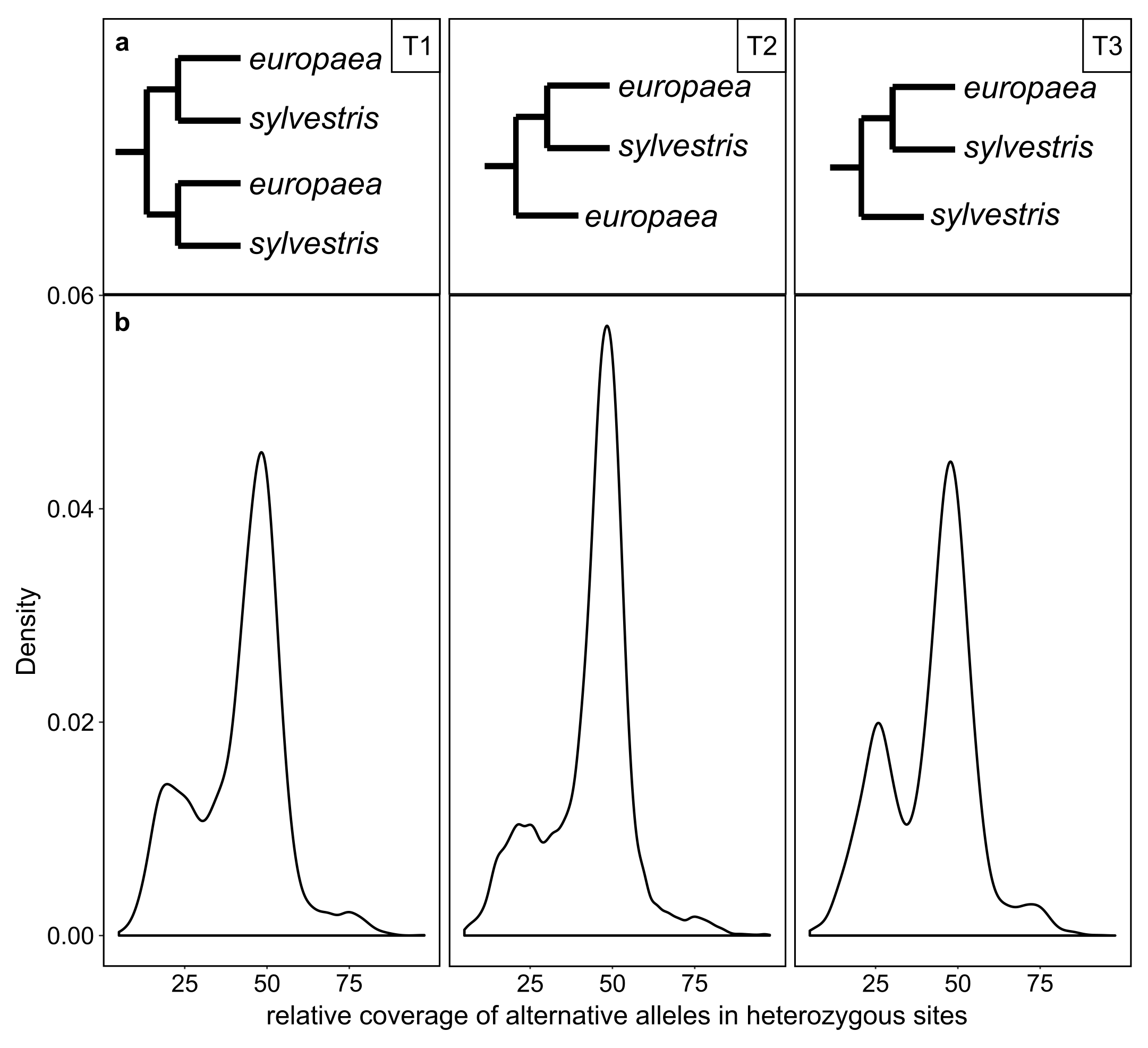

Supplement: Supplementary file 13 — The relative coverage of alternative alleles in heterozygous sites. We assume that we have only two alleles. a In a diploid organism, for the heterozygous positions, we will have one option and, for instance, we will observe one single peak at 0.5. b In a triploid organism, we will have two options for the heterozygous positions (1/3 or 2/3), so in the plot we will observe two peaks at 0.33 and 0.67. c For a tetraploid organism, we will have three options (1/4, 2/4, and 3/4) so we will observe three peaks at 0.25, 0.50, and 0.75. (TIFF 264 kb) [file 12915_2018_482_MOESM13_ESM.tiff]

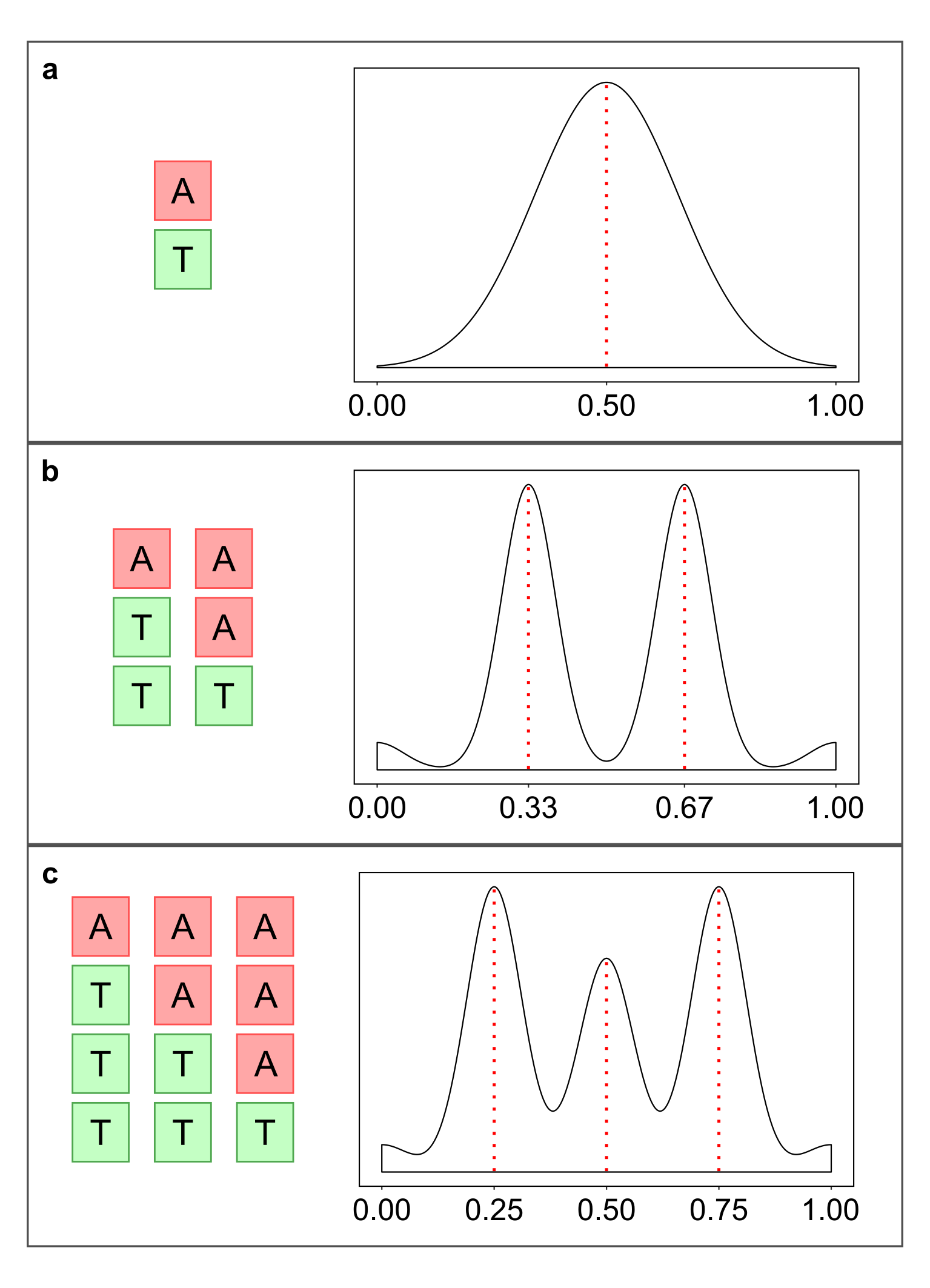

Supplement: Supplementary file 14 — Relative coverage of alternative alleles in heterozygous sites of a tree with different lists of proteins. a Gene tree topologies used to get the olive proteins. T1: A complete gene tree, where both sides conserve both var. europaea and sylvestris. T2: One side of the gene tree has lost the sylvestris copy. T3: One side of the gene tree has lost the europaea copy. b Relative coverage of the alternative alleles in heterozygous sites for each of the gene lists as obtained from the tree gene topologies. (TIFF 493 kb) [file 12915_2018_482_MOESM14_ESM.tiff]
